# Factors Released by Polarized Neutrophil-like Cells Modulate Cardiac Fibroblast Phenotype and Limit the Inflammatory Response After Myocardial Infarction

**DOI:** 10.3390/biomedicines13112829

**Published:** 2025-11-20

**Authors:** Letitia Ciortan, Ana-Maria Gan, Sergiu Cecoltan, Mihaela Serbanescu, Andreea Cristina Mihaila, Razvan Daniel Macarie, Monica Madalina Tucureanu, Miruna Larisa Naie, Mihai Bogdan Preda, Bogdan-Paul Cosman, Galyna Bila, Rostyslav Bilyy, Elena Butoi

**Affiliations:** 1 Inflammation Department, Institute of Cellular Biology and Pathology “Nicolae Simionescu”, 050568 Bucharest, Romania; 2 Stem Cell Biology Department, Institute of Cellular Biology and Pathology “Nicolae Simionescu”, 050568 Bucharest, Romania; 3 Medical and Pharmaceutical BioNanoTechnologies Department, Institute of Cellular Biology and Pathology “Nicolae Simionescu”, 050568 Bucharest, Romania

**Keywords:** neutrophils, cardiac fibroblast phenotype, inflammation, myocardial infarction, neutrophil secretome

## Abstract

**Background**: Following myocardial infarction (MI), cardiac fibroblasts (CFs) adopt distinct phenotypes to ensure scar formation and healing. Although leukocytes are a critical driver of post-MI healing, the role of neutrophils in modulating CF phenotype remains insufficiently explored. We therefore investigated the impact of soluble mediators released by neutrophil subtypes found post-MI—pro-inflammatory (N1) and anti-inflammatory (N2)—on shaping CFs phenotype. **Methods**: In vitro, human 3D grown CFs were indirectly co-cultured with N1 or N2 neutrophil-like cells using a two-chamber Transwell system. After 24 h, expression of inflammatory, remodeling, and pro-fibrotic markers was evaluated in fibroblasts and conditioned media. In vivo, soluble mediators derived from polarized mouse neutrophils (SN1 or SN2) were injected into the infarcted myocardium of C57BL/6J after MI surgery. The effects on the healing process were investigated at 1 and 7 days post-MI. **Results**: In vitro, CFs were found to exhibit a pro-inflammatory and matrix-degrading phenotype following indirect co-culture with N1 cells, characterized by overexpression of IL-1β, IL-6, MCP-1, and metalloproteases MMP-3/MMP-9. In vivo, both SN1 and SN2 treatments significantly reduced pro-inflammatory markers IL-1β and IL-6 gene expression at day 1 post-MI (inflammatory phase). At day 7 post-MI (resolution phase), SN1/SN2 treatments continued to limit local inflammation, while mitigating fibrotic remodeling by reducing CCN2, α-SMA, and key extracellular matrix proteins. **Conclusions**: Together, these findings suggest that while N1-derived mediators promote a pro-inflammatory fibroblast phenotype in vitro, factors secreted by both N1 and N2 support a more balanced reparative response in vivo, by limiting local inflammation and potentially mitigating adverse remodeling post-MI.

## 1. Introduction

Fibroblasts are cells of mesenchymal origin responsible for producing the extracellular matrix (ECM) that surrounds all the cells throughout the body tissues. While essential for tissue development and homeostasis [1], fibroblasts are also involved in the progression of various fibrotic diseases, characterized by excessive production and buildup of ECM proteins [2,3]. Fibrosis is a major contributor to the pathophysiological consequences of myocardial infarction (MI) [4], which is among the leading causes of death and morbidity in the world [5].

Recent evidence reveals substantial heterogeneity in fibroblast populations, both within and across organs, primarily driven by variations in ECM composition [6]. This heterogeneity partly explains their diverse functional roles [7]. In the heart, quiescent cardiac fibroblasts maintain homeostasis but transform into activated myofibroblasts following injury. Intermediate phenotypes also emerge, reflecting the dynamic transitions fibroblasts can undergo in vivo and in vitro, in response to environmental cues [8]. For example, fibroblasts cultured on conventional polystyrene cell culture plates rapidly differentiate into myofibroblasts, producing large amounts of ECM proteins, as we and others have previously shown [9,10]. In contrast, when cultured on specific ECM proteins in 3D cell culture systems, stress fibers disassemble, alpha-smooth muscle actin (α-SMA) expression is markedly reduced, and collagen synthesis is attenuated [9,11].

Following MI, leucocytes massively infiltrate the heart, and cardiac fibroblasts undergo dynamic phenotypic transitions, contributing to the regulation of inflammatory and reparative responses [12]. During the initial inflammatory phase of infarct healing, fibroblasts adopt a pro-inflammatory, matrix-degrading phenotype that contributes to further leukocyte recruitment to the affected area [13]. During the proliferative phase, the clearance of dead cells and matrix debris triggers the activation of anti-inflammatory pathways in phagocytes, resulting in the release of transforming growth factor beta (TGF-β). This promotes the conversion of inflammatory fibroblasts into myofibroblasts, characterized by increased expression of α-SMA [14]. Myofibroblasts rapidly secrete large amounts of ECM proteins to fill the affected area, forming a provisional collagen-based scar to maintain cardiac integrity. During the later stages of scar maturation, myofibroblasts disassemble stress fibers, reduce α-SMA expression, and differentiate into specialized matrifibrocytes involved in scar maintenance [15]. This phenotypic plasticity highlights the central role of fibroblasts in cardiac remodeling and fibrosis.

Recent findings establish neutrophils (polymorphonuclear blood granulocytes) as critical regulators of post-MI repair [16,17], as they massively infiltrate the ischemic region and change their phenotype over time to regulate inflammation resolution and support scar formation. The first study that analyzed the temporal neutrophil polarization post-MI identified only 2 populations, N1 pro-inflammatory, and N2 anti-inflammatory neutrophils [12]. Our transcriptional and functional analysis showed that N1 neutrophils exhibit increased production of inflammatory cytokines, reactive oxygen species (ROS), matrix-degrading enzymes, and enhanced chemotaxis, while N2 neutrophils display upregulated expression of anti-inflammatory markers CD206, Ym1, Arg1 [18]. Recent single-cell RNA sequencing data revealed greater neutrophil heterogeneity post-MI, with five to six major subsets, including SiglecF^hi^ neutrophils. Dominant during the first three days post-MI, SiglecF^hi^ (CD170^hi^) neutrophils are enriched for inflammatory molecules and exhibit heightened ROS production [19,20]. While the exact number of subsets varies, the dichotomy between pro-inflammatory and anti-inflammatory neutrophil phenotypes remains central to their role in inflammation resolution, scar formation, and overall disease progression.

Although the role of neutrophils in the different stages of infarct healing has been previously studied [21], some of the observed effects may be indirect, mediated by secreted factors acting on local cell types, such as fibroblasts. We hypothesized that soluble mediators released by polarized neutrophil subtypes (N1 and N2) differentially regulate cardiac fibroblast activation and ECM remodeling, influencing the balance between inflammation and repair following myocardial infarction.

## 2. Materials and Methods

### 2.1. HL-60 Cell Culture and Polarization into N1 and N2 Neutrophil-like Cells

Promyelocytic human leukemia cells from the HL-60 line (CCL-240) purchased from ATCC were grown in IMDM media (Gibco, New York, NY, USA) supplemented with 20% fetal bovine serum (FBS) (Gibco, New York, NY, USA) and 1% penicillin/streptomycin (P/S) (Thermo Fisher Scientific, Waltham, MA, USA).

Before use in co-culture experiments, HL-60 cells were differentiated into granulocytes following exposure for 5 days to 1.3% DMSO (Sigma Aldrich, St. Louis, MO, USA). The differentiated HL-60 cells (dHL-60, referred as N), which were previously thoroughly characterized for their neutrophil phenotype [22,23,24], were further polarized by exposure for 24 h to specific activators. Pro-inflammatory neutrophil-like cells (referred as N1) were obtained using 20 ng/mL IFN-γ (Sigma Aldrich, St. Louis, MO, USA) and 100 ng/mL LPS (Sigma Aldrich, St. Louis, MO, USA), and anti-inflammatory neutrophil-like cells (referred as N2) were obtained using 20 ng/mL IL-4 (Sigma Aldrich, St. Louis, MO, USA). Immediately before use in co-culture experiments, polarized N1 and N2 cells were washed to remove all traces of the activators and placed in Transwell inserts with 0.4 µm pore size (Corning, New York, NY, USA).

### 2.2. Human Cardiac Fibroblasts (Adult)

Adult human cardiac fibroblasts (CFs) (C-12375) purchased from Merck (Darmstadt, Germany) were sub-cultured using Cardiac Fibroblast Growth Medium (316-500—Cell Applications, San Diego, CA, USA). Cells within passages 2–4 were used to ensure preservation of the fibroblast phenotype.

### 2.3. Hydrogel Preparation

Aortic Root-derived Hydrogel (ARdH) was obtained from fresh porcine aortic roots as previously described in [25]. Briefly, aortic roots were cleaned and decellularized using a detergent/nuclease method. The resulting tissue was lyophilized and digested using porcine intestinal pepsin (Sigma Aldrich, St. Louis, MO, USA) in a 10 mM HCl acid solution until a viscous gel was obtained. The gel was stored at 4 °C for up to 1 month until further use.

### 2.4. The 3D Co-Culture Model

Co-culture between fibroblasts and neutrophil-like cells was conducted for 24 h under indirect (no-contact) conditions—using Transwell systems (Corning, New York, NY, USA) for 6-well plates with 0.4 µm pore size inserts, which prevented transmigration while allowing soluble factor exchange. First, ARdH hydrogel was neutralized with NaOH and kept on ice for 15 min. The mixture was buffered in PBS and centrifuged at 3000× *g*/4 °C for 15 min to remove air bubbles. The resulting gel was used to coat 6-well tissue culture plates in a thin layer, which were incubated for 2 h at 37 °C to allow collagen assembly. Before cell seeding, polymerized ARdH was equilibrated in cell culture media for 1 h. Human CFs were plated on hydrogels at a density of 1.4 × 10^5^ cells/well and allowed to adhere for 24 h. The following day, indirect co-cultures were established by placing the Transwell inserts with N/N1/N2 neutrophil-like cells into wells with human CFs. Cells were left to interact for 24 h in fresh RPMI 1640 media without FBS and P/S. At the end of the experiment, conditioned media was collected, centrifuged at 5000× *g*/4 °C for 10 min, and stored at −20 °C for further use. Human CFs were released from the ARdH hydrogel by enzymatic digestion with Liberase, and were either used to extract protein with RIPA (Thermo Fisher Scientific, Waltham, MA USA) or RNA with Trizol reagent (Ambion, Berlin, Germany).

### 2.5. Generation of N1/N2 Secretome

Neutrophils isolated from healthy mouse bone marrow, as described in Supplementary methods, were plated into 6-well plates (TPP, Transadingen, Switzerland) at a density of 30 × 10^6^ cells/1 mL/well and polarized for 2 h into N1 pro-inflammatory neutrophils using IFN-γ and LPS, and into N2 anti-inflammatory neutrophils using IL-4. In our previous work, we showed that these cells have similar profiles to human N1 inflammatory and N2 anti-inflammatory neutrophils observed in disease states [18].

Following polarization, the cells were pelleted, washed of agonists, and incubated for another 18 h in 1 mL/well of serum-free RPMI media. The next day, conditioned media was harvested and centrifuged to remove cellular debris. The resulting 1 mL supernatant from each well was concentrated 5-fold using centrifugal filters with a 3 kDa cut-off (Amicon Ultra-15, Millipore, Burlington, MA, USA) to a final volume of 200 µL of either SN1 or SN2, and then aliquoted and stored at −80 °C before use. All MI mice received injections from the same pooled and concentrated N1/N2 secretome batch.

### 2.6. Animal Groups and MI Surgical Procedures and SN1/SN2 Treatment

16-week-old C57BL/6 J male mice were bred and housed in the animal facility of ICBP “Nicolae Simionescu”. The study was conducted in accordance with the Declaration of Helsinki. All animal procedures and experiments were performed in accordance with the European Guidelines for Animal Welfare (Directive 2010/63/EU), and approved by the Institutional Ethics Committee of ICBP N. Simionescu (6/9 October 2019) and the National Sanitary Veterinary and Food Safety Authority (591/20 January 2021). All mice were maintained under a 12 h light/dark cycle and had ad libitum access to standard chow and water before and after surgery. For experiments, mice were randomly divided into five groups: (1) 4 Control (C)—unoperated mice, (2) 7 SHAM, (3) 17 Myocardial infarction injected with RPMI—MI, (4) 13 MI injected with N1 secretome (SN1)—MI_SN1, and (5) 14 MI injected with N2 secretome (SN2)—MI_SN2.

### 2.7. MI Surgical Procedure and N1/N2 Secretome Injection

MI was induced by left coronary artery (LCA) ligation, following the surgical procedure previously described by our group [26]. Briefly, mice were administered analgesics and anesthetized by intraperitoneal injection with ketamine (80 mg/kg) and xylazine (10 mg/kg). After confirmation of deep anesthesia by the absence of the pedal reflex, mice were shaved in the neck and thoracic area and placed on a surgical board. The trachea was exposed under a stereomicroscope (Carl Zeiss, Oberkochen, Germany) and a blunt metal catheter was inserted through the mouth and connected to a MiniVent Ventilator (Harvard Apparatus, Holliston, MA, USA). Artificial ventilation (120 strokes/min, 20 mL·kg^−1^·stroke^−1^) was provided throughout the procedure. A thoracotomy was performed to expose the heart through the third intercostal space. For the MI group, the LCA was permanently ligated to induce MI. SHAM mice were treated similarly, except that the needle and thread were passed through the heart, but no ligature was performed. Immediately after LCA ligation, mice with MI were injected into the left ventricle with 10 μL of either SN1, SN2, or RPMI media as vehicle control using a NanoFil Syringe (WPI, Worcester, MA, USA). The thoracic cavity was then closed back together, and mice were placed on a warm pad to assist with recovery. The mice were closely monitored post-operatively, and appropriate measures were implemented to minimize pain and discomfort. Intra and post-surgical mortality of MI mice was approximately 30% (*n* = 44/63).

At 1 day and 7 days following surgery, mice were anesthetized by intraperitoneal injection with ketamine (80 mg/kg) and xylazine (10 mg/kg). 9 MI, 7 MI_SN1 and 6 MI_SN2 mice were sacrificed at day 1, and 8 MI, 6 MI_SN1 and 8 MI_SN2 mice at day 7. After confirmation of deep anesthesia, the heart was surgically exposed and blood was collected on EDTA through heart puncture and centrifuged to collect plasma. Hearts were cleaned of residual blood by perfusion with ice-cold PBS. For immunohistology, whole hearts were fixed with 4% paraformaldehyde (PFA) following perfusion. For RNA extraction, the left ventricle (LV) was isolated by removing the atria and right ventricle with scissors. In the case of C and SHAM mice, the whole LV was immediately snap-frozen in liquid nitrogen and stored at −80 °C for further analysis. Successful MI induction was confirmed intraoperatively by immediate pallor and akinesia of the anterior left ventricular wall. Moreover, the expression of natriuretic peptides ANP and BNP, two peptides that are immediately increased in the acute phase of an MI [27], was significantly increased in infarcted tissue from all MI mice used in experiments (Supplementary Figure S1). For mice with MI, the LV was further cut into two pieces: (1) MI—infarcted area and (2) remote MI—remote area, before freezing in liquid nitrogen and storage at −80 °C.

### 2.8. Elastase Activity Detection in Ex Vivo Mouse Hearts

The FRET-based Hetero-APA probe for neutrophil elastase activity detection [28] was used to detect NE activity in vivo. 10 μM of probe was injected intraperitoneally (i.p.) 60 min prior to the mice being sacrificed. The hearts were washed to remove blood and were imaged using LICORbio Pearl Trilogy Imager (Li-Cor Biosciences, Lincoln, NE, USA) using laser excitation at 685 nm and emission at 700 nm, with a resolution of 85 µm.

### 2.9. RNA Extraction and qPCR

Cells. Isolation of total RNA from cells was performed using Trizol reagent (Ambion, Berlin, Germany) according to the manufacturer’s protocol.

Tissue. Approximately 15 mg of frozen tissue from each sample was homogenized using zirconium beads and a Bioprep 24 homogenizer (Atlantis Biosciences, Singapore, Singapore). Tissue was homogenized for 15 cycles of 30 s at 4000 rpm directly in 1 mL of Trizol reagent, and RNA was further extracted using the standard protocol.

**Figure 1:**
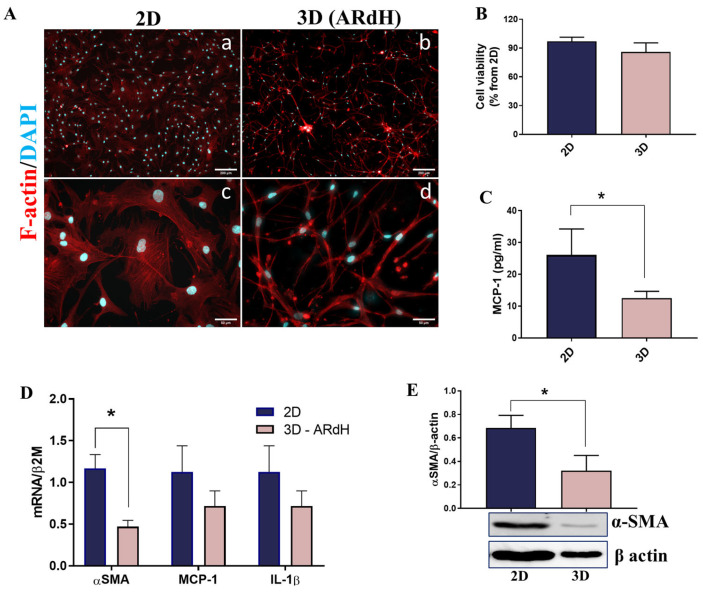
Impact of cell culture conditions on human cardiac fibroblasts (CFs) morphology and phenotype. (**A**) Morphology of CFs after 48h in conventional 2D culture (**a**,**c**) and 3D culture on ARdH hydrogel (**b**,**d**), as determined by F-actin labeling with phalloidin (red) and DAPI nuclear staining (cyan). The scale bar indicates 200 µm for (**a**,**b**), and 50 µm for (**c**,**d**). (**B**) Cell viability of CFs after 48 h in 2D and 3D culture, as determined by MTT assay; (**C**) MCP-1 released in conditioned media by CFs cultured in 2D and 3D conditions determined by ELISA assay; (**D**) mRNA expression of α-SMA, MCP-1, and IL-1β in CFs isolated from 3D-hydrogel or 2D culture after 48 h. (**E**) α-SMA protein expression in CFs from aortic root-derived hydrogel (ARdH) or 2D culture, determined by Western Blot; n = 3, * *p* < 0.05. Data is represented as mean ± SEM.

qPCR. First-strand cDNA synthesis was performed using 1 μg of total RNA and MMLV reverse transcriptase (Invitrogen, Waltham, MA, USA). Amplification of cDNA and quantification of mRNA expression for different genes were performed using the LightCycler 480 Real Time PCR System (Roche Diagnostics, Risch-Rotkreuz, Switzerland) and SYBR Select Master Mix chemistry (Thermo Fisher Scientific, Waltham, MA USA). Primer sequences are shown in Supplementary Materials, Table S1, and relative quantification was performed by comparative CT method The gene expression data were normalized for in vitro experiments to beta2-microglobulin (β2M) as reference gene, and expressed relative to the cardiac fibroblasts grown in 2D conditions (for Figure 1) and to the CFs control group (CFs cultured without neutrophils) for co-culture experiments. Moreover, for in vivo experiments, gene expression data were normalized to the RPL41 housekeeping gene and expressed relative to control, untreated mice. Results are expressed as fold change relative to controls.

### 2.10. Immunohistochemistry

Hearts fixed priorly with 4% PFA were cryopreserved following successive incubations with 10–50% glycerol in phosphate buffer and 6 washes in 3% sucrose. Hearts were further incubated for another hour in OCT compound (Thermo Fisher Scientific, Waltham, MA, USA) before mounting and cryosectioning (5 µm/section). The resulting sections were blocked with PBS with 3% BSA for 1 h and incubated overnight at 4 °C with primary antibodies: anti-mouse Ly6G (cat #127602—BioLegend, San Diego, CA, USA), myeloperoxidase (MPO, cat#AF3667, R&D Systems, Minneapolis, MN, USA), α-SMA (cat#19245, Cell Signaling, Danvers, MA USA). The next day, sections were washed and incubated with secondary: Donkey anti-rabbit 1:1000 (cat #A21207—Cell Signaling, Danvers, MA USA), Goat anti-rat 1:250 (cat #NBP1-75398DL594—Novus Biologicals, Centennial, CO, USA), Donkey anti-goat 1:1000 (cat #A-11058—Cell Signaling, Danvers, MA USA). Nuclei were counterstained with DAPI, and images were acquired using a Leica DMI8 motorized microscope, using a 20× or 40× oil objective.

### 2.11. Protein Extraction and Western Blotting

Cell lysates were prepared by solubilization in RIPA Lysis Buffer (Thermo Fisher Scientific, Waltham, MA, USA) supplemented with a protease inhibitor cocktail according to the manufacturer’s protocol. For immunoblotting, equivalent amounts of extracted proteins were mixed with SX2 and resolved by SDS-PAGE (10% and 15% gels) using a Mini-PROTEAN Tetra Vertical system from BioRad. Transfer to nitrocellulose membranes was performed using a Trans-Blot Semi-Dry system from the same supplier. Membranes were further blocked in Tris-buffered saline (TBS) with 3% bovine serum albumin (BSA) for 1 h and incubated overnight with specific primary antibodies for human IL-1β, matrix metalloproteinase-1 (MMP-1, Cell Signaling, Danvers, MA USA, #54376, 1:1000), MMP-9 (Invitrogen, Waltham, MA, USA, PA5-27191, 1:2000), α-SMA (Cell Signaling, Danvers, MA USA, #19245S, 1:1000), cellular communication network factor 2 (CCN2, Rabbit IgG, Novus Biological, Centennial, CO, USA, NBP2-16026, 1:1000), p38MAPK (Cell Signaling, Danvers, MA USA, #9212, 1:1000), pp38MAPK (Cell Signaling, Danvers, MA USA #4511, 1:1000); ERK1/2 (R&D Systems, Minneapolis, MN, USA, MAB1576, 0.5 µg/mL), pERK1/2 (Cell Signaling, Massachusetts, USA, #4377, 1:1000), and p65 (Invitrogen, Waltham, MA, USA, 51-0500, 1 µg/mL), pp65 (Cell Signaling, Danvers, MA USA, #3033, 1:1000) and housekeeping β-actin (Merck, Darmstadt, Germany, A3854, 1:30,000). Membranes were washed and incubated with secondary HRP-conjugated antibodies. Immunoreactivity was visualized using the SuperSignal™ West Dura Extended Duration Substrate (Thermo Fisher Scientific, Waltham, MA, USA) and the ImageQuant LAS 4000 (GE Healthcare, Chicago, IL, USA) system. Optical density was measured with ImageJ software v1.53c.

### 2.12. Enzyme-Linked Immunosorbent Assay (ELISA)

Secreted levels of human monocyte chemoattractant protein-1 (MCP-1), interleukin-1β (IL-1β), macrophage inflammatory protein (MIP-1α, CCL3), IL-6, MMP-1, MMP-9, TGF-β1, and mouse S100A8/A9, MCP-1, IL-6, and TGF-β1 were measured in conditioned media/mice plasma using DuoSet ELISA kits (R&D Systems, Minneapolis, MN, USA, catalog codes: DY279 (MCP-1), DY201 (IL-1β), DY270 (MIP-1α), DY206 (IL-6), DY901 (MMP-1), DY911 (MMP-9), DY240 (TGF-β1) for human, and DY8596 (S100A8/A9), DY479 (MCP-1), DY406 (IL-6), and DY1679 (TGF-β1) for mouse), according to the manufacturer’s protocol. Briefly, plates were coated overnight with primary antibodies for target molecules at the recommended working concentration. After blocking for 2 h at room temperature, the secondary antibody conjugated with HRP was added. Color development was performed using a tetramethylbenzidine substrate solution, and the reaction was stopped with H_2_SO_4_. Absorbance was measured at 450 nm with correction at 540 nm, and protein levels were calculated using a standard curve.

### 2.13. F-Actin Labelling

Cells were washed twice with PBS and fixed in 4% PFA for 10 min at room temperature. Cells were then permeabilized with 0.2% Triton X-100 for 5 min, and F-actin was labelled using Fluorescein phalloidin (Invitrogen, Waltham, MA, USA) working solution for 20 min at room temperature in the dark. After a washing step, cells were visualized using an Olympus IX81 (Tokyo, Japan) fluorescence microscope equipped with a XM10 camera (Olympus, Tokyo, Japan).

### 2.14. Zymography

Gelatinolytic activity of MMP-2 and MMP-9 was evaluated by zymography in conditioned media from co-cultures. Briefly, conditioned media was mixed with non-reducing Laemmli sample buffer and resolved by SDS-PAGE on 10% gels containing 1 mg/mL porcine skin gelatine. Following electrophoresis, SDS was removed by washing with 2.5% Triton X-100 (2 × 30 min) at room temperature and then the gel was incubated overnight in reaction buffer (50 mmol/L Tris-HCl, pH 7.4, 10 mmol/L CaCl_2_ and 0.2 mmol/L PMSF) at 37 °C. Subsequently, gels were stained with 0.2% Coomassie brilliant blue R-250 and de-stained with 10% acetic acid and 25% methanol. Clear bands of lysis against a blue background indicated the presence of gelatinases. Image acquisition was performed using the ImageQuant LAS 4000 (GE Healthcare, Chicago, IL, USA) system and optical density was measured with ImageJ software v1.53c and expressed as arbitrary units.

### 2.15. DNA Labelling in Plasma

Plasma was diluted 10-fold with HBSS 1X and incubated with 5 μM Sytox Green (Invitrogen, Waltham, MA, USA) for 5 min at RT in the dark. Fluorescence was quantified using an Infinite 200 PRO plate reader (Tecan, Mannedorf, Switzerland)—Ex 485 nm/Em: 527 nm.

### 2.16. Proliferation Assay (xCelligence)

Conditioned media from pro- and anti-inflammatory mouse neutrophils was assessed for its ability to induce fibroblast proliferation using the xCELLigence RTCA DP System (Agilent, Santa Clara, CA, USA). Briefly, E-plates were equilibrated for 30 min with 100 µL/well complete DMEM/F12 fibroblast media, and background impedance was measured. 5 × 10^3^ fibroblasts were seeded in each well, and cells were left to adhere for 24 h. The next day, the media was changed with pre-equilibrated mouse N1/N2 conditioned media, and cells were left in the incubator over 3 days to monitor fibroblast proliferation. Impedance measurements were taken every 15 min during the experiment.

### 2.17. 3-(4,5-Dimethylthiazol-2-Yl)-2,5-diphenyltetrazolium Bromide (MTT) Assay

Cell viability and proliferation were assessed using the TACS^®^ MTT Cell Proliferation Assay (R&D Systems, Minneapolis, MN, USA) according to the manufacturer’s protocol. Briefly, CFs were cultured in either 2D or 3D conditions. After 48 h in culture, MTT reagent was added to each well and the plate was incubated for another 6 h to allow for the formation of insoluble purple formazan dye. Detergent Reagent was then used to solubilize the formazan dye and absorbance was measured at 565 nm.

### 2.18. Statistical Analysis

Student’s *t*-test was used for the comparison between two experimental groups and one-way ANOVA with Tukey’s multiple comparisons when more than two groups were compared. A *p*-value of *p* < 0.05 was considered statistically significant. Data is expressed as the mean of at least three different experiments ± standard error of the mean (SEM). Statistical analysis was performed using GraphPad Prism software version 7.0.

## 3. Results

### 3.1. Cardiac Fibroblasts Exhibit a Quiescent Phenotype When Cultured on a Hydrogel Derived from Native Cardiac ECM

To study the response of fibroblasts to pathological stimuli, it is crucial to first establish a quiescent (non-activated) cell phenotype in vitro. Over the past years, it has become clear that traditional 2D cell culture in monolayers is inadequate for this purpose, as substrate rigidity is known to promote fibroblast-to-myofibroblast transition (FMT) [29]. To more accurately replicate the in vivo environment and maintain a quiescent cell phenotype, human CFs were grown in a hydrogel derived from native cardiac ECM (ARdH) that we have recently developed [25]. First, we investigated the morphology, proliferation, and phenotype of CFs cultured on this hydrogel compared to CFs maintained in 2D culture. F-actin staining revealed that human CFs from conventional 2D culture exhibited signs of activation, including a larger cell body and actin filaments assembled into stress fibers (Figure 1A(a,c)). In contrast, CFs grown in 3D conditions on ARdH displayed an elongated spindle-shaped body and formed a complex cell network distributed within the hydrogel without exhibiting visible stress fibers (Figure 1A(b,d)). Cell proliferation was slightly reduced (approximately 10%) in 3D compared with 2D conditions (Figure 1B). Gene and protein expression of myofibroblast marker α-SMA was found to be approximately 2-fold and 3-fold higher in CFs maintained in 2D compared to 3D conditions, respectively (Figure 1D,E). Furthermore, although the gene expression of inflammatory mediators IL-1β and MCP-1 was not significantly reduced in CFs grown in 3D versus 2D culture (Figure 1D), the ELISA assay showed a 2.1-fold decrease (*p* = 0.0486) of secreted MCP-1 in the conditioned media from CFs grown on ARdH versus 2D conditions (Figure 1C). Soluble IL-1β level was also evaluated, but was either absent or below the sensitivity threshold of the assay (250 pg/mL). Together, these results show that 3D culture using the hydrogel promotes a quiescent phenotype of human CFs and provides a better in vitro model to study the phenotypic transition of CFs in response to pathological stimuli. Therefore, this culture system was used in all subsequent experiments presented in this study.

### 3.2. Cardiac Fibroblasts Acquire a Pro-Inflammatory and Matrix-Remodeling Phenotype, with Reduced Proliferative Capacity upon Exposure to Soluble Mediators Released by Pro-Inflammatory Neutrophil-like Cells

Before starting co-culture experiments with human CF, the gene expression profile and degranulation status of dHL60-neutrophil-like cells polarized towards N1 and N2 were evaluated. The results confirmed that human dHL60 exposed to IFN-γ/LPS exhibit increased levels of N1 pro-inflammatory markers TNF-α, IL-1β, CCL3, chemokine C-C motif ligand (CCL5), and intracellular cell adhesion molecule-1 (ICAM-1) when compared with control or IL-4 exposed cells (Supplementary Figure S2A). Moreover, they presented increased amounts of released granular proteins, including MMP-9, MPO, and cytokine/chemokines, as compared with control or N2 cells (Supplementary Figure S2B). The influence of mediators released from these pro-/anti-inflammatory neutrophil-like cells on cardiac fibroblast phenotype was then assessed via indirect co-culture of CF with dHL-60 (N) and polarized dHL-60 (N1, N2) cells.

**Figure 2:**
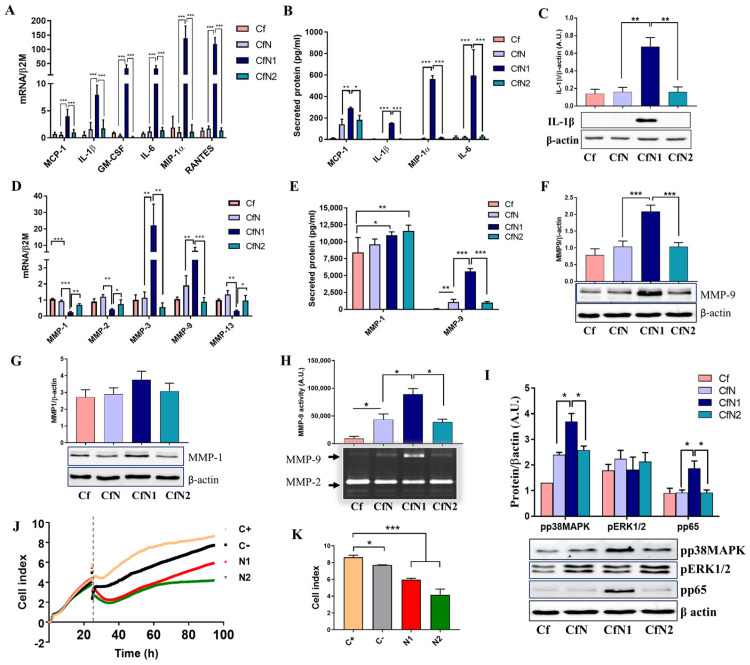
Profile of inflammatory mediators and of matrix metalloproteinases (MMP) following a 24 h indirect co-culture of human CFs grown on ARdH hydrogel with control granulocytes (CfN), or their pro-inflammatory (N1) or anti-inflammatory (CfN2) subtypes. (**A**) mRNA level of inflammatory cytokines and chemokines in CFs grown on ARdH hydrogel and indirectly interacted with granulocytes subsets, determined by qPCR—normalized to B2M and represented as fold change compared with C (Cf only); (**B**) Levels of secreted inflammatory cytokines and chemokines in conditioned media from indirect co-cultures as evaluated by ELISA assay; (**C**) IL-1β protein level in CFs lysates following indirect interaction with neutrophil-like cell subtypes, as determined by Western Blot assay and normalized to β-actin; (**D**) Metalloproteases MMP-1, -2, -3, -9 and -13 mRNA expression in CFs indirectly co-cultured with neutrophil-like subtypes; (**E**) Concentration of soluble MMP-1 and MMP-9 quantified in conditioned media as evaluated by ELISA assay; (**F**,**G**) Protein expression of MMP-9/-1 in CFs following indirect interaction, as evaluated by Western Blot and normalized to β-actin; (**H**) The enzymatic activity of gelatinase MMP-9 in the conditioned media assessed by SDS-PAGE gelatine zymography; (**I**) Intracellular signaling: MAPKs (p38 and ERK1/2) and NF-κB phosphorylation in human CFs following indirect interaction with neutrophil-like cell subtypes; (**J**) Proliferative capacity of primary mouse CFs assessed using the xCelligence system: C+—fibroblasts exposed to DMEM/F12 with 10%FBS and C-—fibroblasts exposed to DMEM/F12 without FBS; N1/N2—fibroblasts exposed to mouse N1/N2 conditioned media; (**K**) Cell index after ~3 days of exposure to N1/N2 conditioned media. n = 3, * *p* < 0.05, ** *p* < 0.01, *** *p* < 0.001 Data is represented as mean ± SEM.

Gene expression analysis revealed that mRNA levels of inflammatory cytokines and chemokines in fibroblasts co-cultured with unpolarized N (CfN) or anti-inflammatory N2 cells (CfN2) are similar to levels of control cardiac fibroblasts (non-interacted, Cf). In contrast, CFs co-cultured with N1 pro-inflammatory cells (CfN1) presented a significant 5-, 11-, 48- and 45-fold increase in MCP-1, IL-1β, GM-CSF and IL-6 mRNA, respectively (Figure 2A). Additionally, exposure to N1 neutrophil-like cells induced significant expression of MIP-1α and RANTES mRNAs in human CFs, showing 193- and 164-fold increases in CfN1, respectively, compared to the control group (Figure 2A). Soluble and intracellular levels of MCP-1, MIP-1α, IL-1β, and IL-6 were assessed in conditioned media and human CFs lysates. Results showed that interaction of fibroblasts with all granulocytes subtypes led to the release of soluble MCP-1 in the media when compared with control fibroblasts, but interaction with N1 cells produced nearly double the level of secreted MCP-1 (290.5 ± 6.404 pg/mL) compared to CFs interacted with N (140.5 ± 27.7 pg/mL) or N2 (181.9 ± 24.38 pg/mL) cells. Moreover, significant amounts of soluble IL-1β, MIP-1α, and IL-6 were detected in the media only following CFs interaction with N1 cells (Figure 2B). Western Blot analysis confirmed IL-1β protein expression exclusively in lysates from CFs co-cultured with N1 cells, with no detectable expression in control or CFs co-cultured with N or N2 cells (Figure 2C).

In addition to the increased production of inflammatory mediators, the activated fibroblast phenotype is also characterized by altered expression of matrix-degrading metalloproteinases [15]. Therefore, we next investigated the effect of indirect co-culture with polarized neutrophil-like cell subtypes on MMP regulation in human CFs. Gene expression analysis revealed that MMP-1, -2, and -13 mRNA were significantly decreased, and MMP-3 and -9 significantly increased in CFs co-cultured with N1 neutrophil-like cells, compared with CFs co-cultured with N or N2 cells (Figure 2D). ELISA assay performed on conditioned media showed that MMP-1 was secreted by both control and interacting cells. However, the concentration of MMP-1 secreted by control Cf (8426 ± 1268 pg/mL) was significantly lower than that in media from Cf co-cultured with N1 (10969 ± 220.6 pg/mL) and N2 cells (11605 ± 382.9 pg/mL). MMP-9 was undetectable in the media of non-interacted Cf but was present following co-culture with N, N1, or N2 cells. Notably, communication with N1 cells led to a substantial ~5-fold increase in secreted MMP-9 levels (5608 ± 216.2 pg/mL) compared to the co-culture with N (1076 ± 205.1 pg/mL) or N2 (989.5 ± 82.15 pg/mL) cells (Figure 2E). Similarly, MMP-9 protein expression was doubled in CfN1 compared to CfN or CfN2 (Figure 2F), and MMP-1 protein levels also showed a slight increase upon interaction with N1 cells; however, this difference was not statistically significant (Figure 2G). Importantly, the elevated concentration of MMP-9 protein in conditioned media from human CFs exposed to N1 cells was paralleled by a marked increase in MMP-9 enzymatic activity. While interaction with all neutrophil-like cell subtypes enhanced MMP-9 activity in the conditioned media, the activity level was approximately twice as high following indirect co-culture of CFs with N1 cells compared to those that interacted with N or N2 cells (Figure 2H). MMP-2 activity was not significantly modified between the samples (Supplementary Figure S3A).

Cardiac fibroblasts possess complex intracellular signaling mechanisms that enable them to coordinate both inflammatory and fibrotic responses. Therefore, co-culture of human CFs with N1 cells significantly increased the phosphorylation of p38MAPK (mitogen-activated protein kinases) and activation of the p65 subunit of NF-κB transcription factor. Phospho-p65 protein level was found to be twice as high as in CfN1 compared to CfN or CfN2, which presented a similar pp65 level as control, non-interacted CFs. ERK1/2 phosphorylation, however, remained unaffected following co-culture with either N1 or N2 subtype (Figure 2I).

Functional analysis of primary mouse fibroblast proliferation in response to conditioned media from mouse BM-derived N1 and N2 neutrophils showed that mouse CFs exhibit impaired proliferative capacity following exposure to mouse N1/N2 secretome (Figure 2J,K).

### 3.3. Pro-Inflammatory Neutrophil-like Cells Impair the Transition of Cardiac Fibroblasts to Myofibroblasts

To obtain a complete image of the impact of neutrophils on human CFs phenotype, we further investigated the potential of different neutrophil-like cell populations to modulate the expression of several key molecules associated with the myofibroblast phenotype in CFs. Previous quantitative proteomic studies indicated that most of the protein content of the human myocardium is made up of various fibrillar collagen components [30]. Among these, type I and type III are the primary collagens synthesized and deposited by myofibroblasts during homeostasis, as a result of wound healing, and also in fibrotic diseases, where the excessive collagen deposition leads to organ dysfunction and failure [31].

**Figure 3:**
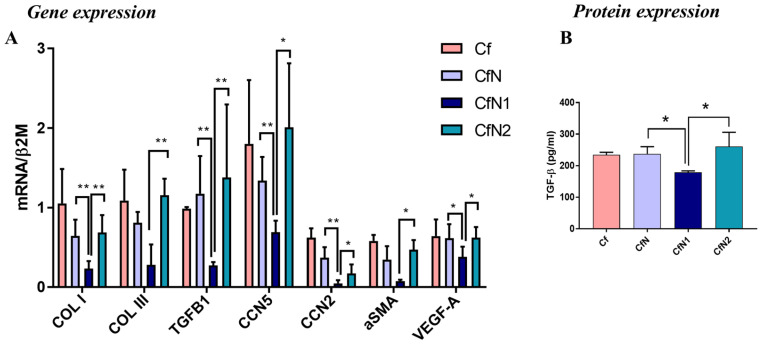
The impact of granulocyte subtypes on the expression of molecules associated with fibrosis in human CFs following 24 h indirect co-culture with neutrophil-like cell subsets. (**A**) mRNA of fibrotic markers in CFs grown on ARdH hydrogel and indirectly interacted with granulocyte subsets, determined by qPCR assay—normalized to β-actin and represented as fold change compared with control Cf (non-interacted); (**B**) Concentration of soluble TGF-β1 quantified in conditioned media following indirect interaction as evaluated by ELISA assay; n = 3, * *p* < 0.05, ** *p* < 0.01. Data is represented as mean ± SEM.

Our results showed that following a 24 h interaction with pro-inflammatory N1 cells, human CFs strongly down-regulate the gene expression of master fibrosis regulator TGF-β1 and myofibroblast marker α-SMA compared to control non-interacted Cf (*p* < 0.0001 and *p* = 0.0024) or CfN2 (*p* = 0.0277 and *p* = 0.0106) (Figure 3A). Protein analysis further revealed a significant decrease in soluble TGF-β1 in the conditioned media following interaction with N1 neutrophil-like cells (Figure 3B), but not of α-SMA in CFs lysates (Supplementary Figure S3B). These changes were also accompanied by a marked decrease in collagen I and III mRNA expression in CfN1 (0.23- and 0.28-fold change) compared to CfN (0.64- and 0.81-fold change) or CfN2 (0.69- and 1.15-fold change) (Figure 3A). Moreover, VEGF mRNA expression was also downregulated in CFs following co-culture with N1, but not with N or N2 neutrophil-like cells (Figure 3A). These findings are consistent with a previous RNA-seq study of fibroblast gene signatures post-MI, which demonstrated that only fibroblasts from day 3, characterized by a proliferative phenotype, showed upregulated VEGFA gene expression. In contrast, fibroblasts on day 1 post-MI exhibited a pro-inflammatory profile with a leukocyte-recruiting gene signature and showed downregulated TGF-β signaling [32].

The CCN family consists of cell/ECM-associated proteins with diverse roles in modulating different cell functions in response to environmental factors. Expression of two members of the CCN family with important roles in fibrosis, CCN2 (or CTGF-connective tissue growth factor, pro-hypertrophic and pro-fibrotic molecule) and CCN5 (anti-hypertrophic and anti-fibrotic molecule) [33] was found to be altered following co-culture experiments. Thus, CCN5 and CCN2 mRNA was significantly down-regulated in CfN1 when compared to control (Cf), CfN, or CfN2 (Figure 3A). Western Blot results also show a decrease in CCN2 protein level, but without statistical significance (Supplementary Figure S3C).

### 3.4. Administration of N1 and N2 Secretome in the Infarcted Area Diminishes the Inflammatory Response at Day 1 Post-MI

**Figure 4:**
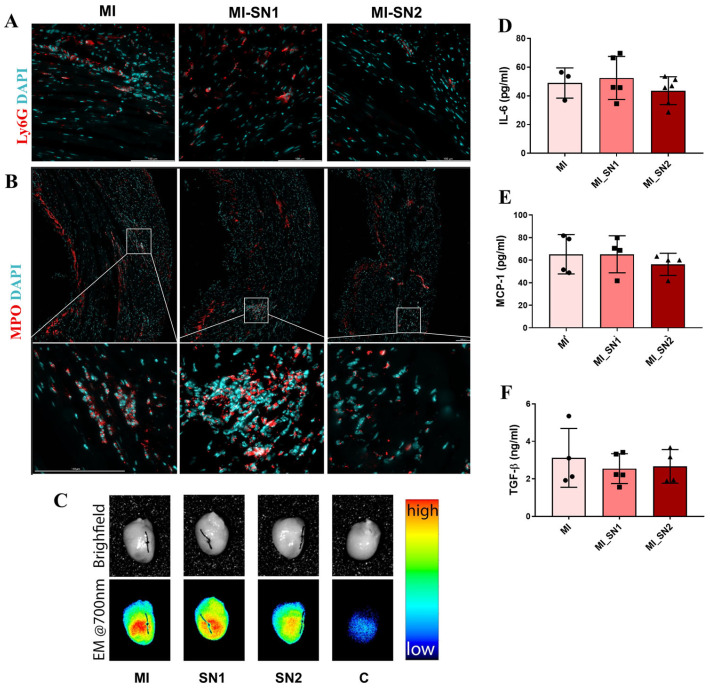
Effect of N1/N2 secretome (SN1/SN2) in modulating the inflammatory phase—day 1 post-MI. (**A**,**B**) Immunofluorescence of Ly6G (red) and MPO (red) in cardiac tissue from MI mice with or without treatment with SN1/SN2, 1-day post-MI. Nuclei are counterstained with DAPI (cyan). Scale bar always represents 100 μm. (**C**) Neutrophil elastase activity detected directly in explanted mouse hearts at 1-day post-MI; (**D**–**F**) Levels of IL-6, MCP-1, and TGF-β1, respectively, in plasma from MI mice treated or not with SN1/SN2, as evaluated by ELISA. n ≥ 3. Data is represented as mean ± SD.

The impact of soluble mediators released by neutrophil subtypes on cardiac fibroblast phenotype and MI progression was further investigated using a mouse model of MI, which received an injection with secretome from either N1 or N2 neutrophils in the infarcted area immediately after artery ligature. To validate the MI model, we investigated neutrophil-associated markers in the infarcted area and plasma at 24 h post-MI. Therefore, the presence of elastase—a protease abundant in neutrophil primary granules and bound by released neutrophil extracellular traps (NETs), and Ly6G, a granulocyte surface marker, were present in the left ventricle of MI and SHAM mice, but absent in unoperated mice, demonstrating the presence of infiltrated neutrophils (Supplementary Figure S4A,B). Moreover, the plasma levels of MCP-1 and S100A8/A9—key markers linked to N1 neutrophils [16] were significantly elevated in surgically treated mice (SHAM and MI) compared to controls. From these groups, mice with MI had significantly increased S100A8/A9 concentrations (*p* = 0.0059) and slightly higher MCP-1 concentration in their plasma, as compared with SHAM mice (Supplementary Figure S4C,D). Moreover, neutrophil systemic activation was indirectly assessed by measuring plasma DNA as a marker of NET release, using Sytox Green staining. Results showed that mice with acute MI (24 h) exhibit significantly higher plasma DNA concentrations compared to SHAM (*p* = 0.0032) and control (*p* = 0.0002) mice (Supplementary Figure S4E). The effects of N1 and N2 secretome in modulating the initial inflammatory phase (day 1 post-MI) were assessed both systemically (in plasma) and locally (in the heart). Plasma analysis revealed no significant change in IL-6, MCP-1, and TGF-β1 levels between MI and MI treated with either SN (Figure 4D–F). Similarly, IHC results revealed the presence of Ly6G and MPO, in the infarcted area from all MI mice (Figure 4A,B). Furthermore, neutrophil recruitment to the left ventricle following MI was confirmed by the increased neutrophil elastase activity compared to unoperated control mice (C), detected in situ using the FRET-based Hetero-APA probe (Figure 4C).

**Figure 5:**
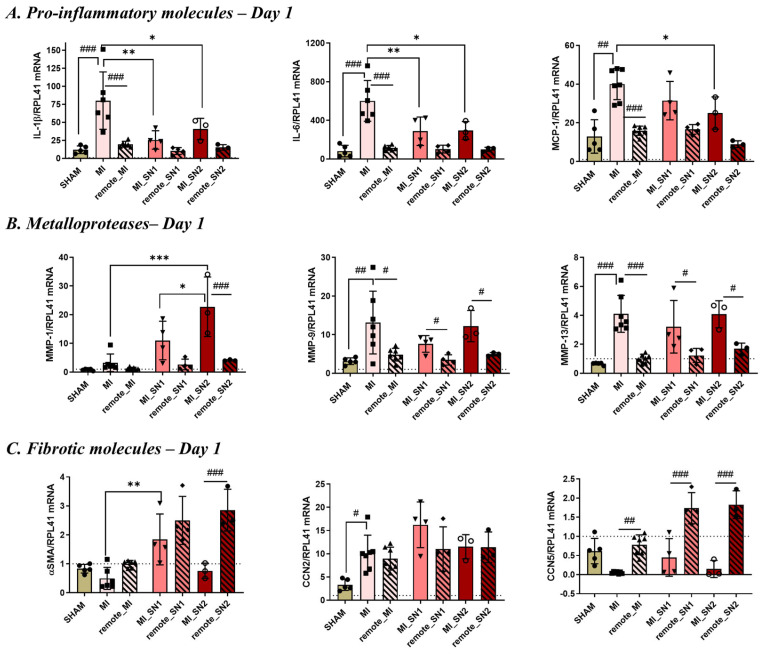
Gene expression of pro-inflammatory, matrix remodeling and fibrotic mediators at day 1 post-MI in left ventricle heart tissue. (**A**) Pro-inflammation markers IL-1β, IL-6 and MCP-1; (**B**) Metalloproteases MMP-1, -9, and -13, (**C**) Fibrotic molecules CCN2, CCN5, and α-SMA, quantified in LV tissue of SHAM, MI mice with or without SN1/SN2, as well as in the corresponding remote area from each MI mouse. Gene expression data were generated by qPCR, normalized to RPL41 housekeeping gene, and expressed relative to control—untreated mice (dotted line). n = 5/6 mice per group, * MI vs. MI with SN1/SN2, # MI tissue vs. corresponding remote tissue from each MI mouse. ^#,^ * *p* < 0.05, ^##,^ ** *p* < 0.01, ^###,^ *** *p* < 0.001. Data is represented as mean ± SD.

Gene expression of inflammatory and profibrotic molecules was quantified in the left ventricle of SHAM, MI, and MI mice treated with either SN1 or SN2. The qPCR data showed that IL-1β, IL-6, and MCP-1, but also MMP-9, MMP-13 and CCN2 mRNA, were significantly increased at day 1 in the infarcted (MI) region compared to the remote area or SHAM heart tissue (Figure 5), consistent with an active inflammatory response at this time point. Surprisingly, both SN1 and SN2 treatments were found to downregulate the inflammatory response following MI, while also modulating distinct aspects of early post-MI tissue remodeling.

Specifically, SN1 significantly reduced the expression of pro-inflammatory cytokines IL-1β and IL-6, and simultaneously increased the expression of α-SMA, a marker associated with myofibroblast activation (Figure 5A,C). In addition, SN1 treatment (but not SN2) upregulated the gene expression of well-established pro-fibrotic markers α-SMA and CCN2 at day 1, indicating early myofibroblast activation. Although the increase in CCN2 expression did not reach statistical significance in our study, this may reflect the limited sample size rather than a lack of biological relevance, as previous studies have demonstrated that myofibroblast activation assessed by α-SMA expression is significantly impaired in the absence of CCN2 in vitro [34]. The increased expression of these two molecules may accelerate tissue repair but could also lead to excessive ECM deposition. In contrast, SN2 treatment led to a broader anti-inflammatory effect by significantly downregulating IL-1β, IL-6, and MCP-1, while selectively enhancing the expression of MMP-1, a matrix-remodeling enzyme (Figure 5A,B). Increased MMP-1 expression on day 1 in response to SN2 may indicate an accelerated collagen degradation process in the damaged tissue, a crucial step for normal healing, which could facilitate the rapid clearance of necrotic debris, thereby promoting a faster tissue repair response.

The TGF-β gene expression was not found to be significantly different between the animal groups (Supplementary Figure S4F).

### 3.5. Factors Released by N1/N2 Neutrophils Reduce FMT and ECM Deposition at 7 Days Post-MI

**Figure 6:**
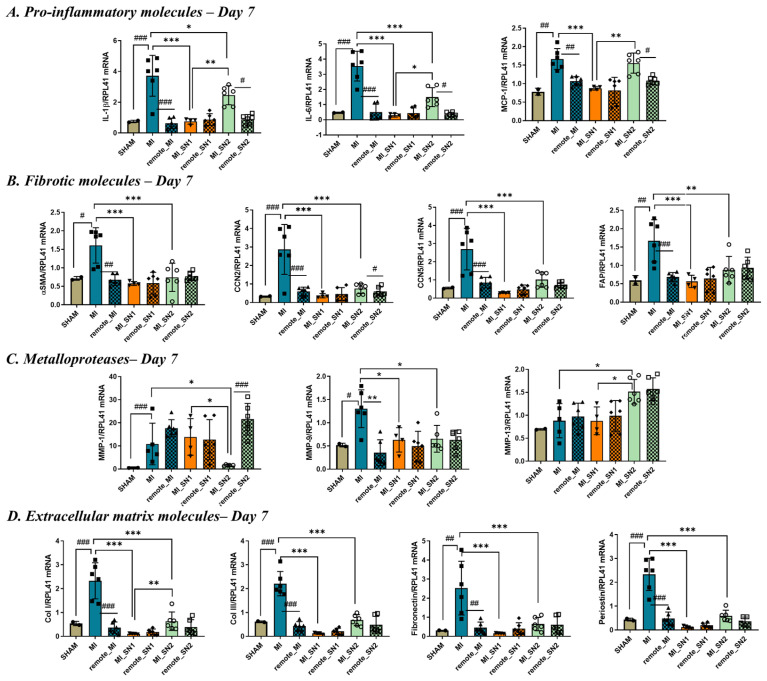
Gene expression of pro-inflammatory, matrix-degrading, fibrotic and ECM remodeling mediators at day 7 post-MI in LV heart tissue. (**A**) Pro-inflammatory molecules IL-1β, IL-6 and MCP-1; (**B**) Fibrotic molecules α-SMA, CCN2, CCN5, and FAP; (**C**) Metalloproteases MMP-1, -9, and -13; and (**D**) Extracellular matrix molecules proteins COL I, COL III, Fibronectin and Periostin, quantified in LV tissue of SHAM, mice with MI with or without SN1/SN2, as well as in the corresponding remote area from each MI mouse. Gene expression data were generated by qPCR and normalized to healthy control mice. n = 5/6 mice per group, * MI vs. MI with SN1/SN2, # MI tissue vs. corresponding remote tissue from each MI mouse. ^#,^ * *p* < 0.05, ^##,^ ** *p* < 0.01, ^###,^ *** *p* < 0.001. Data is represented as mean ± SD.

To evaluate the impact of neutrophil secretome administration during the reparative phase of myocardial infarction healing, the molecular markers previously evaluated at day 1 post-MI were re-examined at day 7 following SN1/SN2 injection. As expected, all inflammatory markers were markedly reduced in MI tissue by day 7 compared to day 1, consistent with the natural resolution of the acute inflammatory response (Supplementary Figure S5A). Similar to day 1, MI mice treated with SN1 or SN2 continued to exhibit lower expression of inflammatory genes compared to untreated MI mice at day 7 (Figure 6A), suggesting a sustained anti-inflammatory effect of both secretomes. The profibrotic gene CCN2, known to drive ECM deposition and scar formation [35], was highly expressed in all MI mice as early as day 1 and remained elevated in the infarcted region at day 7, reflecting ongoing fibrotic remodeling (Supplementary Figure S5C). Notably, SN1 and SN2 treatment significantly reduced CCN2 expression at day 7 compared to untreated MI mice (Figure 6B), indicating their potential to attenuate pathological fibrosis. Conversely, CCN5, an anti-fibrotic and anti-hypertrophic factor [35], was significantly downregulated in the infarct region at day 1 post-MI but partially recovered by day 7 in untreated MI mice—possibly as a compensatory response to increased fibrosis (Figure 6B). In line with this, α-SMA, a hallmark of FMT, which was increased in SN1 mice at day 1, was significantly increased in untreated MI mice by day 7, reflecting activation of myofibroblasts. Mice that received SN1 or SN2 injections presented with significantly reduced α-SMA expression at this stage (Figure 6B).

Given the observed impact of secretome treatment on post-MI remodeling, we further examined the expression of key ECM molecules at day 7 post-infarction. As expected, all ECM-related genes—including collagen I, collagen III, fibronectin, and periostin—were significantly upregulated in MI mice, reflecting active fibrotic remodeling. Importantly, treatment with either SN1 or SN2 led to a marked reduction in the expression of these ECM components (Figure 6D), indicating that both secretomes effectively attenuate excessive matrix deposition.

**Figure 7:**
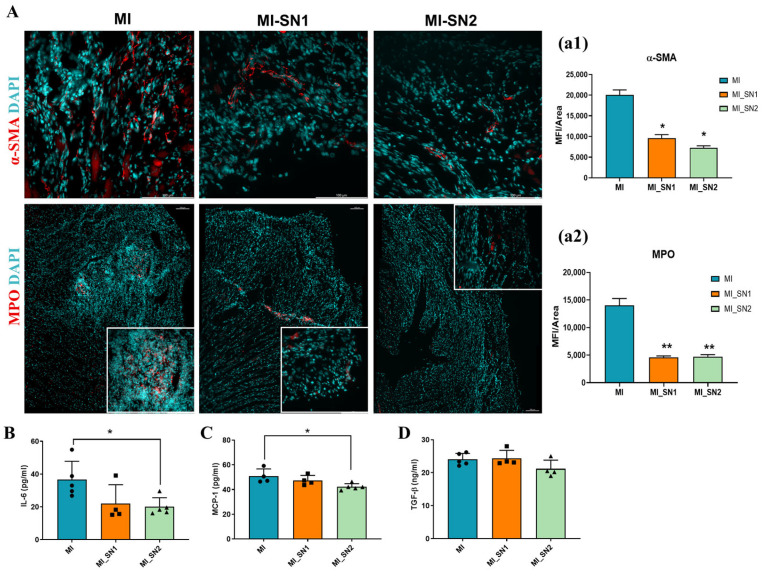
Effect of N1/N2 secretome (SN1/SN2) in the reparative phase—7 days post-MI. (**A**) Immunofluorescence staining of α-SMA (red) and MPO (red) in mouse cardiac tissue from MI mice with or without treatment with SN1/SN2. Nuclei are counterstained with DAPI (cyan). Scale bar always represents 100 μm; Signal intensity was quantified within the infarcted zone, selected from slide scans acquired at 20× magnification, and expressed as mean fluorescence intensity (MFI) per infarct area (**a1**,**a2**). (**B**–**D**) Levels of IL-6, MCP-1 and TGF-β1, respectively, in plasma from mice with MI, treated or not with SN1/SN2, as evaluated by ELISA assay. n ≥ 3, * *p* < 0.05, ** *p* < 0.01. Data is represented as mean ± SD.

Immunofluorescence analysis revealed that α-SMA and MPO expression levels were reduced by more than 50% in SN1- and SN2-treated mice compared to untreated MI animals (Figure 7A). This reduction in myofibroblast and inflammatory cell markers at 7 days post-MI aligns with gene expression data, suggesting that BM neutrophil-derived secretome may inhibit the fibroblast-to-myofibroblast transition within the infarct zone, thereby mitigating fibrotic remodeling. At this time point, in addition to attenuating local inflammation, treatment with neutrophil secretome also reduced plasma levels of pro-inflammatory markers IL-6 and MCP-1, with a significant value for MI-mice treated with SN2 (Figure 7B,C). TGF-β plasmatic level exhibited a modest reduction only in MI mice treated with SN2, but with no statistical significance (Figure 7D).

## 4. Discussion

Following MI, cardiac fibroblasts undergo distinct phenotypic transitions in response to dynamic environmental cues and orchestrate inflammation resolution, scar formation, and tissue repair, with critical impact on cardiac recovery. These transitions include a proinflammatory state, a matrix-synthetic phenotype, and ultimately, they acquire a quiescent phenotype during the maturation phase of healing [36]. Among the various factors influencing these transitions, neutrophils and their secreted mediators are likely key drivers of fibroblast phenotypic switching.

In the present study, in vitro data revealed that soluble mediators released by N1 neutrophil-like cells promote a pro-inflammatory fibroblast phenotype and impair fibroblast proliferation and migration. In vivo data showed that treatment of the LV with secretome derived from polarized neutrophils modulates the early inflammatory response post-MI, and exerts significant effects during the reparative phase of cardiac healing.

Previous in vitro studies have consistently demonstrated fibroblast plasticity, with cell culture conditions profoundly impacting cell behavior [10,37]. While traditional 2D culture systems provide valuable information, 3D models more accurately recapitulate aspects of the in vivo microenvironment and provide biologically relevant insights [38]. To achieve this, our study employed a 3D hydrogel ARdH, which we recently developed and showed that promotes a quiescent phenotype of valvular interstitial cells [25]. CFs cultured on this hydrogel were also shown to exhibit a quiescent phenotype, marked by reduced expression of the myofibroblast marker α-SMA and inflammatory molecules MCP-1 and IL-1β. Using a double-chamber co-culture system, CFs grown on 3D hydrogels were allowed to exchange soluble molecules with neutrophil-like cells polarized into N1 and N2 subtypes, with the study primarily focusing on the pro-inflammatory and pro-fibrotic molecules produced by fibroblasts. Our in vitro findings demonstrated that factors released by N1 cells significantly induced pro-inflammatory markers such as IL-1β, IL-6, MCP-1, RANTES, and MIP-1α, as well as metalloproteases MMP-1 and MMP-9 in fibroblasts, driving them toward a pro-inflammatory and matrix-degrading phenotype. Moreover, soluble mediators released by pro-inflammatory N1 cells significantly impaired fibroblast proliferation and migration (Supplementary Figure S6). These findings are consistent with previous studies, demonstrating that alarmins released by necrotic cells promote pro-inflammatory and matrix-degrading fibroblast phenotypes during the inflammatory phase of infarct healing [15]. Moreover, they provide new evidence that, in addition to alarmins, factors released by N1 neutrophil-like cells further contribute to driving the shift toward a pro-inflammatory fibroblast phenotype.

In vitro, fibroblast phenotype changes induced by indirect cross-talk with N1 cells involved p38 MAPK activation and NF-κB signaling. MAPKs were previously associated with fibroblast phenotype switching, and Mapk14 (p38α) deletion was shown to block myofibroblast differentiation in vivo [39]. Similarly, NF-κB activation was found in a subset of CFs from mice subjected to pressure overload, resulting in overexpression of MCP-1 [40], a chemotactic protein also found upregulated in our in vitro and in vivo results. Targeting NF-κB in activated CFs has been shown to reduce Ly6C^hi^ monocyte recruitment and preserve cardiac function [40]. Given that N1 neutrophils degranulate and secrete extracellular vesicles or a diverse range of inflammatory mediators [18], it is plausible that some of them (such as IL-1β, S100A9, S100A12, NETs) bind to fibroblasts, triggering cellular signaling and NF-κB activation. Among these, IL-1β is a strong candidate due to its established role in driving fibroblast activation into a pro-inflammatory and matrix-degrading phenotype. Previously, it was found that IL-1β, signaling through IL-1R1, induces inflammatory activation of fibroblasts and impairs myofibroblast differentiation and proliferation [41]. Similarly, S100A8/A9 or S100A12, known to directly interact with cell surface receptors such as TLR4 and RAGE, activate different signaling pathways, including NF-κB [42]. Consistent with these mechanisms, our in vitro data demonstrated that TLR4 signaling mediates fibroblast activation, as evidenced by reduced gene expression of IL-1β, IL-6, TNF-α, and MMP-9 when a TLR4 inhibitor (CLI-095) was used (Supplementary Figure S7). These findings align with a broader concept of fibroblast plasticity following MI, where fibroblasts undergo dynamic transitions to regulate inflammatory, reparative, and angiogenic responses [13]. Increased levels of inflammatory cytokines post-MI delay FMT, allowing sufficient time for debris clearance and tissue remodeling [41]. Our results support this paradigm, showing that pro-fibrotic molecules, including collagens and TGF-β1, were significantly downregulated in CFs exposed to the pro-inflammatory environment produced by N1.

In vivo data using a mouse MI model further support these findings and reveal an exacerbated inflammatory status in the infarcted area during the early phase (day 1) of MI, when N1 neutrophils are known to be the predominant neutrophil subtype found in high numbers in the necrotic area [12]. This intense inflammatory response was accompanied by increased expression of MMPs, indicative of a matrix-degrading environment in the infarcted region. Surprisingly, both SN1 and SN2 treatments exerted comparable anti-inflammatory effects at day 1 post-MI, attenuating early post-infarction inflammation as reflected by the reduced expression of IL-1β and IL-6. However, their downstream effects diverged: SN1 increased α-SMA expression, suggesting promotion of early myofibroblast activation, while SN2 downregulated MCP-1 and enhanced MMP-1 expression, suggesting a role in modulating inflammation and matrix remodeling.

By day 7 post-MI, inflammatory gene expression declined across all MI groups (Supplementary Figure S5), reflecting the expected resolution of inflammation [12]. Since inflammatory markers and MMPs were globally assessed in the injured tissue, without isolating specific cell populations, they likely reflect contributions from infiltrating leukocytes and activated fibroblasts. However, after 7 days, the myofibroblast marker α-SMA and CCN2, molecules expressed only by fibroblasts, were highly elevated in the infarcted area but not in the adjacent area or SHAM mice, suggesting the existence of a fibrotic process. These data align with prior RNAseq data showing a dynamic fibroblast response during MI, with a pro-inflammatory, leukocyte-recruiting, pro-survival, and anti-migratory phenotype on the first day post-MI [32]. By day 3, they shift to a proliferative and reparative state, marked by increased expression of pro-fibrotic and pro-angiogenic markers [32].

While the anti-inflammatory response induced by SN1 and SN2 is vital during the early inflammatory phase (day 1), after 7 days, only residual inflammation persists, as all investigated inflammatory markers were dramatically reduced in all groups, including MI group (Supplementary Figure S5A). In this context, an important difference between SN1 and SN2 treatments lies in the modulation of MCP-1 at day 1, which was significantly reduced in SN2 but not in SN1 group. This is particularly relevant since MCP-1 has been identified as an independent prognostic value in the acute and chronic phases after ACS [43]. Moreover, in a large cohort of patients with acute coronary syndromes, elevated baseline MCP-1 levels were independently associated with increased risk for death or recurrent MI [44]. Another noteworthy difference between the two treatments is the elevated MMP-1 expression observed at day 1 in response to SN2, which may indicate increased collagen degradation within the damaged tissue, leading to enhanced clearance of necrotic debris and accelerated tissue repair. Notably, this assumption is further supported by the marked reduction in MMP-1 expression by day 7 in mice treated with SN2, suggesting that matrix degradation is largely resolved by this stage. In contrast, SN1-treated mice maintained MMP-1 levels comparable to those observed in MI mice (approximately 15-fold higher than in sham mice), indicating a prolonged phase of ECM remodeling. Both secretomes reduced the expression of CCN2 and α-SMA (at day 7), indicating suppression of myofibroblast activation. Additionally, they downregulated key ECM components—including collagen I, collagen III, fibronectin, periostin, and FAP—suggesting inhibition of excessive matrix deposition. SN2 further reduced MMP-1 and MMP-9, whereas SN1 selectively decreased MMP-9, highlighting their distinct regulatory effects on ECM turnover. These molecular changes were accompanied by cellular proliferation within the necrotic area observed at day 7 in all MI groups, with a slight increase in Ki67 expression following SN2 treatment (Supplementary Figure S8). This may indicate a more pronounced beneficial effect of SN2 compared to SN1 in promoting post-infarction repair.

Previous data showed that neutrophils play a crucial role in cardiac repair after MI by polarizing macrophages toward a reparative phenotype [45]. This mechanism may be responsible, at least in part, for the beneficial effects we observed following treatment with SN2, as we recently demonstrated the capacity of SN2 to increase macrophage efferocytosis [46]. The impact of SN1 and SN2 on leukocyte infiltration into the infarcted myocardium may also help limit local inflammation, representing a potential mechanism underlying the beneficial effects observed following treatment. Additionally, the N1/N2 secretome exhibited limited NETs levels as compared with activated neutrophils undergoing NETosis (Supplementary Figure S9). Therefore, local injection of soluble factors released by N1 and N2 may promote tissue repair without eliciting the detrimental effects commonly associated with excessive NET formation, which has been previously linked to poor outcomes in MI patients [47]. This is also supported by decreased expression of inflammatory cytokines and reduced MPO and neutrophil elastase (NETs markers) in the left ventricle of treated mice, suggesting attenuated inflammatory cell recruitment and less secondary tissue damage. A key factor potentially contributing to these effects is IL-10, which we identified in the secretome of N1 neutrophils [18]. IL-10 is a well-established anti-inflammatory cytokine known to suppress pro-inflammatory signaling, limit neutrophil activation, and promote resolution of inflammation. Its presence in SN1 may help explain the sustained anti-inflammatory environment observed in vivo and the reduction in fibrotic remodeling. However, further studies are needed to delineate the specific role of IL-10 and other secreted mediators in orchestrating the protective effects of SN1 and SN2 during post-MI healing. In addition, the apparent contrasting in vitro and in vivo findings regarding the impact of N1 soluble factors on modulating the inflammatory response provide a hint of the intricate cellular interplay and regulatory complexity that occurs in the MI microenvironment.

While these findings are compelling, the limitation of our study lies in: (i) the use of HL-60-derived neutrophil-like cells and bone marrow-derived murine neutrophils for in vitro data, which may not fully capture the phenotypic diversity and temporal dynamics of neutrophil subpopulations observed in vivo following MI; (ii) in vivo: the relatively small number of animals per group, primarily due to the technical complexity and high mortality associated with the MI model, as well as ethical considerations regarding animal use. Therefore, subtle differences in the expression of some molecules (such as CCN2) between SN1- and SN2-treated groups could be underestimated. Additionally, the study only focused on two time points—day 1 and day 7 post-MI—which represent the acute inflammatory and early remodeling phases, respectively. However, intermediate time points, such as day 3 post-MI, may provide important insights into the temporal dynamics of neutrophil secretome effects, particularly during the transition from inflammation to repair.

## 5. Conclusions

These data suggest that while the in vitro setting shows the direct impact of neutrophil-secreted factors on fibroblasts, highlighting their anti-fibrotic and anti-proliferative effects, the in vivo context reflects a more dynamic interaction involving immune modulation, local cytokine milieu, and temporal regulation of fibroblast phenotypes. Early in vivo responses may reflect an initial compensatory activation of fibroblasts, but the sustained downregulation of pro-fibrotic and pro-inflammatory pathways by day 7 aligns with the in vitro prediction of a long-term anti-fibrotic influence of neutrophil secretome. Together, these findings highlight the pivotal role of neutrophil-derived factors in coordinating the spatial and temporal dynamics of post-MI repair. The observed benefits of neutrophil secretomes—particularly their ability to reduce inflammation and limit adverse remodeling—underscore their therapeutic potential. Considering that trans-endocardial injection is a safe and feasible approach in MI patients [48], these secretomes may represent a promising strategy to enhance myocardial healing and improve clinical outcomes after infarction.

## Data Availability

Data from our research is available from the corresponding authors upon reasonable request.

## Supplementary Materials

The following supporting information can be downloaded at: https://www.mdpi.com/article/10.3390/biomedicines13112829/s1, Table S1: The sequences of oligonucleotide primers (human and mouse) used to evaluate gene expression; Supplementary methods: Wound healing assay, Proteome profiler cytokine array, Mouse primary cardiac fibroblast isolation, Mouse primary bone marrow-derived neutrophils, Co-culture model; Figure S1: Gene expression of the natriuretic peptides ANP and BNP in LV tissue from SHAM and MI mice (± SN1/SN2 injection) at 1-day post-MI, normalized to healthy control mice that did not undergo surgical procedures, n = 4/5 mice per group; * *p* < 0.01, ** *p* < 0.01, *** *p* < 0.001, (Sham vs. MI/MI_SN1/MI_SN2); Figure S2: Transcriptomic and proteomic profile of polarized N1/N2 neutrophils derived from differentiated HL-60 cells (dHL60). (A) Gene expression of pro-inflammatory molecules TNFα, IL-1β, CCL3, CCL5, and ICAM-1 in dHL60 control (Ctrl), or polarized for 18h with LPS+IFNγ (for N1 pro-inflammatory phenotype) and IL-4 (for N2 anti-inflammatory phenotype). n = 3, ** *p* < 0.01, *** *p* < 0.001, (LPS+IFNγ vs. Ctrl or IL-4); (B) Neutrophil degranulation molecules quantified in the conditioned media from polarized dHL-60 cells by cytokine array. Figure S3: (A) The enzymatic activity of gelatinases MMP-2 in the conditioned media of human CFs following indirect interaction with N/N1/N2 neutrophils, assessed by SDS-PAGE gelatine zymography; (B,C) Protein expression of myofibroblast marker α-SMA and pro-fibrotic matricellular protein CCN2 in human CFs following indirect interaction with neutrophils, as assessed by Western Blot and normalized to β-actin; n = 3 Data is represented as mean ± SEM; Figure S4: Local and systemic markers of neutrophil activation in acute (day 1) MI. (A) Representative images of neutrophil elastase (green) in mouse cardiac tissue from control (a), SHAM (b), or mice with MI (c). DNA is stained red. Scale bar is 50 µm for images a, b, and c, and 10 µm for images c1–c3. The c1–c3 images show an enlarged area from image c where infiltrated neutrophils and potential NETs are visible (white arrows). (B) Immunofluorescence staining of neutrophil marker Ly6G (red) in mouse cardiac tissue from control, SHAM, or mice with MI. Nuclei are stained with DAPI (blue). (C,D) Plasma levels of S100A8/A9 and MCP-1 in the serum of control, SHAM, or mice with MI after 1 day, as evaluated by ELISA assay. (E) DNA presence in serum of control, SHAM, or mice with MI after 1 day, after fluorescent staining with Sytox Green. n = 4–6, ** *p* < 0.01, *** *p* < 0.001. Data is represented as mean ± SD. (F) TGF-β1 mRNA expression in LV tissue from SHAM and MI mice (± SN1/SN2 injection) at 1-day post-MI, normalized to healthy control mice that did not undergo surgical procedures, n = 5/6 mice per group; Figure S5: Gene expression of pro-inflammatory, ECM remodelling and fibrotic mediators during the acute phase (day 1) and the reparatory phase (day 7) post-MI. (A) Pro-inflammatory molecules IL-1β, IL-6, and MCP-1; (B) Metalloproteases MMP-1, -9, and -13; and (C) Fibrotic mediators αSMA, CCN2, and CCN5, quantified in LV tissue of SHAM, mice with MI with or without SN1/SN2. Gene expression data were generated by qPCR and normalized to healthy control mice that did not undergo surgical procedures. n = 5/6 mice per group, * 1 day vs. 7 days, # vs. SHAM. * *p* < 0.05, ** *p* < 0.01, *** *p* < 0.001. Data is represented as mean ± SD; Figure S6: Fibroblast migration in the presence of N1 soluble derived mediators. Representative images from in vitro scratch wound healing assays showing that mouse fibroblast migration is impaired by exposure to factors released by pro-inflammatory mouse BM derived neutrophils (N1 secretome) as compared with fibroblasts exposed to culture media (Ctrl) after 24h (T_24_); Figure S7: Gene expression of pro-inflammatory molecules expressed by mouse cardiac fibroblasts following interaction with mouse BM neutrophils. (A–D) Gene expression of inflammatory markers IL-1β, IL-6, TNFα, and metalloprotease MMP-9 in mouse cardiac fibroblasts—control (cFb), upon co-culture with neutrophils (FbN) or co-culture with neutrophils in the presence of a TLR4 inhibitor (CLI-095). n = 3, ** *p* < 0.01, *** *p* < 0.001, (cFb vs. FbN1) and # *p* < 0.05, ## *p* < 0.01, ### *p* < 0.001 (FbN1 vs. FbN1+CLI); Figure S8: Effect of N1/N2 secretome (SN1/SN2) on cell proliferation in the reparative phase—7 days post-MI. (A) Immunofluorescence staining of Ki67 (red) in mouse cardiac infarcted tissue from MI mice with or without treatment with SN1/SN2. Nuclei are counterstained with DAPI (cyan). Scale bar represents 50 μm; (B) Signal intensity was quantified within the entire infarcted zone, selected from slide scans acquired at 20× magnification, and expressed as mean fluorescence intensity (MFI) per infarct area (a1, a2). Data is represented as mean ± SD; Figure S9: DNA as a marker of NETs presence in human PMN supernatant. Sytox green staining of DNA released in conditioned media of control (unstimulated) human PMN (N), and human PMN cultivated in the presence of N1 (LPS+IFN) and N2 (IL-4) agonists after 2.5 h in culture. n = 3, ** *p* < 0.01, *** *p* < 0.001. Data is represented as mean ± SD control. References [49,50,51] are cited in the Supplementary Materials.

## Abbreviations

The following abbreviations are used in this manuscript:

α-SMA	Alpha-smooth muscle actin
APA	Alternative polyadenylation
ARdH	Aortic root derived hydrogel
Arg1	Arginase 1
B2M	Β2-Microglobulin
BM	Bone marrow
CCN2	Cellular communication network factor 2
CD	Cluster of differentiation
CF	Cardiac fibroblast
DAPI	4′,6-diamidino-2-phenylindole
dHL-60	Differentiated HL-60 cells
DMSO	Dimethyl sulfoxide
ECM	Extracellular matrix
EDTA	Ethylenediaminetetraacetic acid
ELISA	Enzyme-linked immunosorbent assay
FAP	Fibroblast activation protein
FBS	Fetal bovine serum
FMT	Fibroblast-to-myofibroblast transition
FRET	Fluorescence resonance energy transfer
HRP	Horseradish peroxidase
IFN-γ	Interferon gamma
IHC	Immunohistochemistry
IL-	Interleukin-
LCA	Left coronary artery
LPS	Lipopolysaccharide
LV	Left ventricle
MAPK	Mitogen-activated protein kinase
MCP-1	Monocyte chemoattractant protein-1
MFI	Mean fluorescence intensity
MI	Myocardial infarction
MMP	Matrix metalloproteinases
MPO	Myeloperoxidase
NETs	Neutrophil extracellular traps
OCT	Optimal cutting temperature
P/S	Penicillin/streptomycin
PBS	Phosphate-buffered saline
PCR	Polymerase chain reaction
PFA	Paraformaldehyde
RIPA	Radio-immunoprecipitation Assay
ROS	Reactive oxygen species
RT	Room temperature
SDS-PAGE	Sodium dodecyl sulfate–polyacrylamide gel electrophoresis
SN1	Supernatant derived from N1 polarized BM neutrophils
SN2	Supernatant derived from N2 polarized BM neutrophils
TBS	Tris-buffered saline
TGF-β	Transforming growth factor beta
