# Supplementary material for: Factors Released by Polarized Neutrophil-like Cells Modulate Cardiac Fibroblast Phenotype and Limit the Inflammatory Response After Myocardial Infarction
Source: Biomedicines. 2025 Nov 20;13(11):2829. doi: 10.3390/biomedicines13112829 (PMC12650109; doi:10.3390/biomedicines13112829)
Supplement: Supplementary file 1 [file biomedicines-13-02829-s001.zip › biomedicines-3959402-supplementary.docx]

Factors released by polarized neutrophil-like cells modulate cardiac fibroblast phenotype and limit the inflammatory response after myocardial infarction

Letitia Ciortan^1,^*, Ana-Maria Gan^1,^*, Sergiu Cecoltan^1^, Mihaela Serbanescu^1^, Andreea Cristina Mihaila^1^, Razvan Daniel Macarie^1^, Monica Madalina Tucureanu^1^, Miruna Larisa Naie^1^, Mihai Bogdan Preda^2^, Bogdan-Paul Cosman^2^, Galyna Bila^3^, Rostyslav Bilyy^3^ and Elena Butoi^1,^*

^1^ Inflammation Department, Institute of Cellular Biology and Pathology “Nicolae Simionescu”, Bucharest, Romania; secretariat@icbp.ro

^2^ Stem Cell Biology Department, Institute of Cellular Biology and Pathology “Nicolae Simionescu”, Bucharest, Romania; secretariat@icbp.ro

^3^ Medical and Pharmaceutical BioNanoTechnologies Department, Institute of Cellular Biology and Pathology “Nicolae Simionescu”, Bucharest, Romania; secretariat@icbp.ro

***** Correspondence: E.B.: elena.dragomir@icbp.ro; L.C.: letitia.ciortan@icbp.ro; A-M.G: anca.gan@icbp.ro

**Supplementary material**

**Supplementary Tables**:

**Supplementary Table S1.** The sequences of oligonucleotide primers (human and mouse) used to evaluate gene expression:

**Table S1.** The sequences of oligonucleotide primers (human and mouse) used to evaluate gene expression:

| **Gene  (Human)** | **Sequences of Oligonucleotide Primers** | **GenBank accession number** |
| --- | --- | --- |
| αSMA | ACTGCCTTGGTGTGTGACAA | NM_007392 |
|  | CACCATCACCCCCTGATGTC |  |
| MCP-1 | ATTACTTAAGGCATAATGTTTCACA | NM_002982 |
|  | AGCATGAAAGTCTCTGCCGCCCTTCTG |  |
| IL-1β | AGAGTGGAGCCTGGTCTTACA  CCTTTGCTGACAATAAGCACTGG | NM_000450 |
| GM-CSF | TCCTGAACCTGAGTAGAGACAC  TGCTGCTTGTAGTGGCTGG | NM_000758 |
| IL-6 | CTGCAGAATTCCAGGACCACA  TCCGGTGGTGTAAAGAGGAC | XM_054358145 |
| MIP-1alpha | AGTTCTCTGCATCACTTGCTG  CCGCTTCGCTTGGTTAGGAA | NM_002983 |
| RANTES | CCAGCAGTCGTCTTTGTCAC  CTCTGGGTTGGCACACACTT | NM_002985 |
| COL1A2 | AATTGGAGCTGTTGGTAACGC  CACCAGTAAGGCCGTTTGC | NM_000089 |
| COL3A1 | AGGTCCTGCGGGTAACACT  ACTTTCACCCTTGACACCCTG | NM_000090 |
| CCN2 | AAAAGTGCATCCGTACTCCCA  CCGTCGGTACATACTCCACAG | NM_001901 |
| CCN5 | CCCCAGTTTTCTGGCCTTGT  GAAGCGGTTCTGGTTGGACA | NM_001323370 |
| TGF-β | CAAGCAGAGTACACACAGCAT  TGCTCCACTTTTAACTTGAGCC | NM_000660 |
| MMP-1 | AAAATTACACGCCAGATTTGCC  GGTGTGACATTACTCCAGAGTTG | NM_002421 |
| MMP-2 | TACAGGATCATTGGCTACACACC  GGTCACATCGCTCCAGACT | NM_004530 |
| MMP-3 | AGTCTTCCAATCCTACTGTTGCT  TCCCCGTCACCTCCAATCC | NM_002422 |
| MMP-9 | AGACCTGGGCAGATTCCAAAC  CGGCAAGTCTTCCGAGTAGT | NM_004994 |
| MMP-13 | ACTGAGAGGCTCCGAGAAATG  GAACCCCGCATCTTGGCTT | NM_002427 |
| **Gene  (Mouse)** | **Sequences of Oligonucleotide Primers** |  |
| IL-1β | GAAATGCCACCTTTTGACAGTG | NM_008361 |
|  | TGGATGCTCTCATCAGGACAG |  |
| IL-6 | TACCACTTCACAAGTCGGAGGC | NM_031168 |
|  | CTGCAAGTGCATCATCGTTGTTC |  |
| MCP-1 | TAAAAACCTGGATCGGAACCAAA | NM_011333 |
|  | GCATTAGCTTCAGATTTACGGGT |  |
| MMP-1a | AGTATGGGCTGTTCAAGAGCAGAG  TGCTAGGGAAGCCAAAGAAACTG | NM_032006 |
| MMP-9 | GGGGCGTGTCTGGAGATTCG  GGAAACTCACACGCCAGAAGA | NM_013599 |
| MMP-13 | AGCAAGCCAGAATAAAGACTGTGC  AACATGGTGGAGCACAAAGGAG | NM_008607 |
| CCN2 | CCACCCGAGTTACCAATGACA  GTGCACCATCTTTGGCAGTG | NM_010217 |
| CCN5 | GCCCCAGGAGAATACAGGTG  TGACAAGGGCAGAAAGTTGGT | NM_016873 |
| αSMA | TGACTCACAACGTGCCTATCT  CTCACGCTCGGCAGTAGTC | NM_007392 |
| Col1a1 | AAGGGTCATCGTGGCTTCTC  ACCGTTGAGTCCGTCTTTGC | NM_007742 |
| Col3a1 | TCCTGGCAACCCTGGAATAG  GACCTCGTGCTCCAGTTAGC | NM_009930 |
| Fibronectin | GGCTTTGGCAGTGGTCATTTC  TCCCACTTCTCTCCGATCTTG | NM_010233 |
| Periostin | CCATTGGAGGCAAACAACTCC  TTGCTTCCTCTCACCATGCA | NM_015784 |
| FAP | GGCTGGGGCTAAGAATCCG  GCATACTCGTTCACTGGACAC | NM_007986 |

**Supplementary methods**:

*Wound healing assay*

Mouse cardiac fibroblasts were grown to a 100% confluency and afterward, they were deprived of serum for 48 hours to ensure their arrest in the G0 phase of the cell cycle. Next, they were incubated with the secretome of N1 neutrophils, and a lesion in the monolayer (a ”scratch”) was created using a sterile 10 μL pipette tip [49]. The control was represented by fibroblasts cultivated with media plus 1% FBS. Images of the wound were captured with a fluorescence microscope (Olympus IX81) equipped with a XM10 camera, at T_0_ and T_24_ hours post-treatment to assess the migration of fibroblasts over 24 hours. The photos were analyzed using the ImageJ software, delimitating the area of the lesion created with the pipette tip. The speed of migration of the two experimental groups of fibroblasts (with or without the N1 secretome) was calculated as a ratio between the distance migrated and the time fibroblasts were incubated with N1 secretome/1% FBS.

*Proteome profiler cytokine array*

Cytokine profiles of dHL-60 cells (N) and dHL-60 cells polarized into N1/N2 were assessed using the Proteome Profiler Human XL Cytokine Array (ARY022B, R&D Systems) according to the manufacturer’s instructions. Conditioned media collected after 48 h of polarization were processed and incubated with array membranes, which were then washed and incubated with a cocktail of biotinylated detection antibodies, followed by Streptavidin-HRP. The chemiluminescent detection was performed using a Luminescent Image Analyzer LAS-3000 (FUJIFILM, Japan), and mean pixel densities of all spots were determined using CLIQS 1D Pro software (TotalLab, United Kingdom).

*Mouse primary cardiac fibroblast isolation*

16 weeks old C57BL/6 J male mice bred and housed in the animal facility of the Institute of Cellular Biology and Pathology (ICBP) “Nicolae Simionescu” were anesthetized by intraperitoneal injection with ketamine (80 mg/kg) and xylazine (10 mg/kg). After confirmation of deep anesthesia, the thoracic cavity was opened to expose the heart. The left atrium was cut and the heart was perfused with ice-cold PBS for 5 minutes to wash the vasculature. CFs were isolated from mouse hearts using a modified version of a previously described protocol [50]. Briefly, the atria were removed and the ventricles were cut into small pieces using scissors. The resulting tissue was digested using a mixture of Liberase™ DH (Roche, Switzerland) and trypsin for 20 minutes at 37ºC and the supernatant was collected, inactivated with serum, and kept on ice. Another seven digestion steps (10 minutes each) were performed until the tissue pieces were visibly digested. All the collected fractions were pooled together and centrifuged. Cells were plated and left to adhere overnight in complete DMEM/F12 (Gibco, USA) media supplemented with 10% FBS, 1% P/S, 10 μg/ml insulin (Lonza, Switzerland) and 10 ng/μl FGF (Gibco, USA). The next day, adhered fibroblasts were washed of remaining debris, and fresh media was added. Cells were used within the first 4 passages.

*Mouse primary bone marrow-derived neutrophils*

Tibias and femurs were collected from the same mice used to isolate cardiac fibroblasts and cleaned of the remaining tissue. Both ends of the bones were cut, and BM cells were flushed. Neutrophils were isolated by Percoll gradient centrifugation as previously described [18]. Isolated cells were placed on Transwell inserts (Corning, USA) and polarized for 2h into N1 pro-inflammatory neutrophils, using IFN-γ and LPS, and into N2 anti-inflammatory neutrophils using IL-4. Following polarization, the cells were washed of polarizing agonists and used for indirect co-culture experiments with primary mouse cardiac fibroblasts.

*Co-culture model*

Co-culture between mouse primary fibroblasts and BM neutrophils was conducted for 24h under indirect (no-contact) conditions—using Transwell systems (Corning, USA) for 6-well plates with 0.4 µm pore size inserts, which prevented transmigration while allowing soluble factor exchange [51]. Cells were left to interact for 24h in fresh RPMI 1640 media without FBS and P/S. At the end of the experiment, fibroblasts were used to extract RNA with Trizol reagent (Ambion, Germany).

**Supplementary Figures**:

**Supplementary Figure S1.** Gene expression of the natriuretic peptides ANP and BNP in LV tissue from SHAM and MI mice (± SN1/SN2 injection) at 1-day post-MI, normalized to healthy control mice that did not undergo surgical procedures, n=4/5 mice per group; *p<0.01, **p<0.01, ***p<0.001, (Sham vs. MI/MI_SN1/MI_SN2).


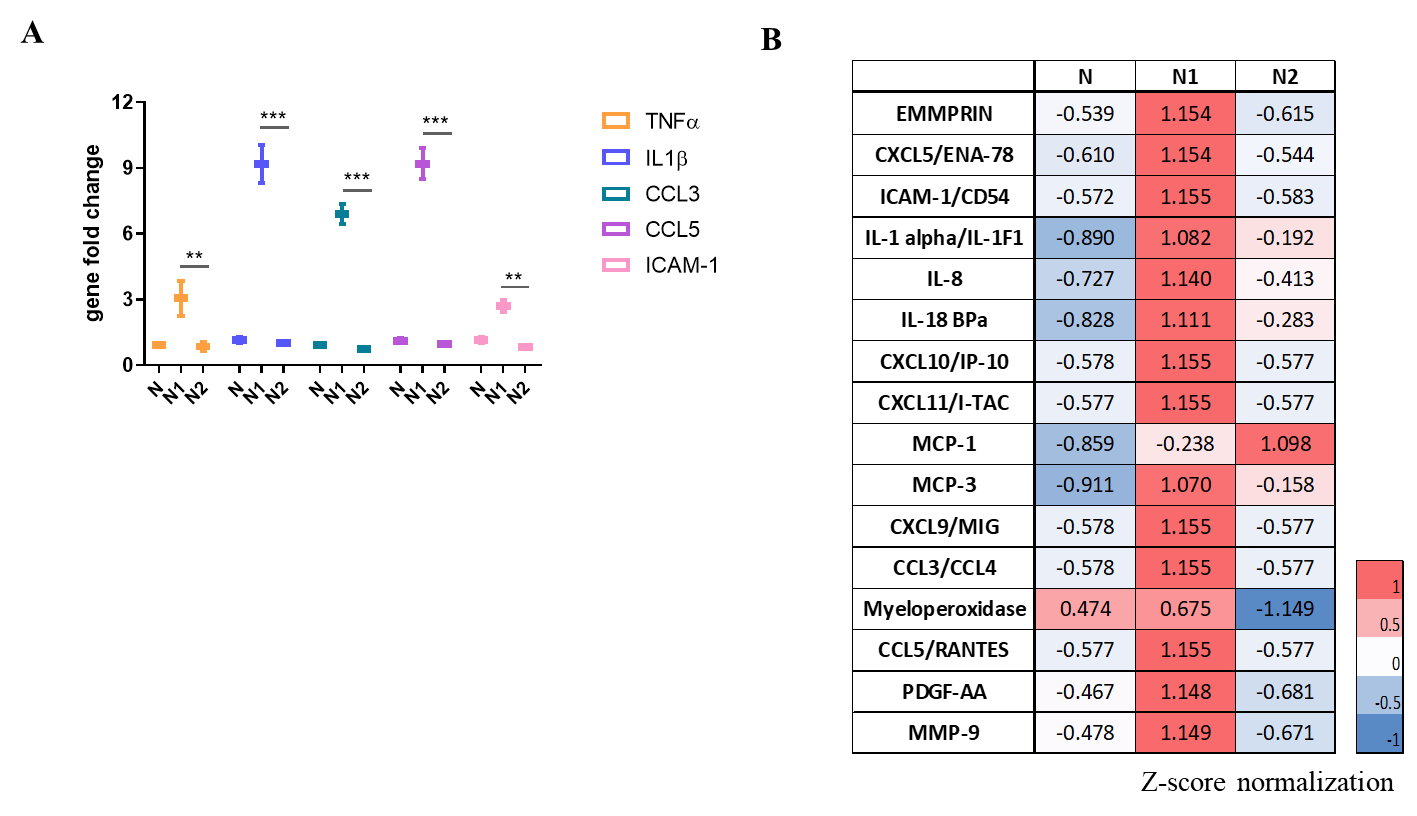


**Supplementary Figure S2.** Transcriptomic and proteomic profile of polarized N1/N2 neutrophils derived from differentiated HL-60 cells (dHL60). (**A).** Gene expression of pro-inflammatory molecules TNFα, IL-1β, CCL3, CCL5, and ICAM-1 in dHL60 control (Ctrl), or polarized for 18h with LPS+IFNγ (for N1 pro-inflammatory phenotype) and IL-4 (for N2 anti-inflammatory phenotype). n=3, **p<0.01, ***p<0.001, (LPS+IFNγ vs. Ctrl or IL-4); (**B).** Neutrophil degranulation molecules quantified in the conditioned media from polarized dHL-60 cells by cytokine array.


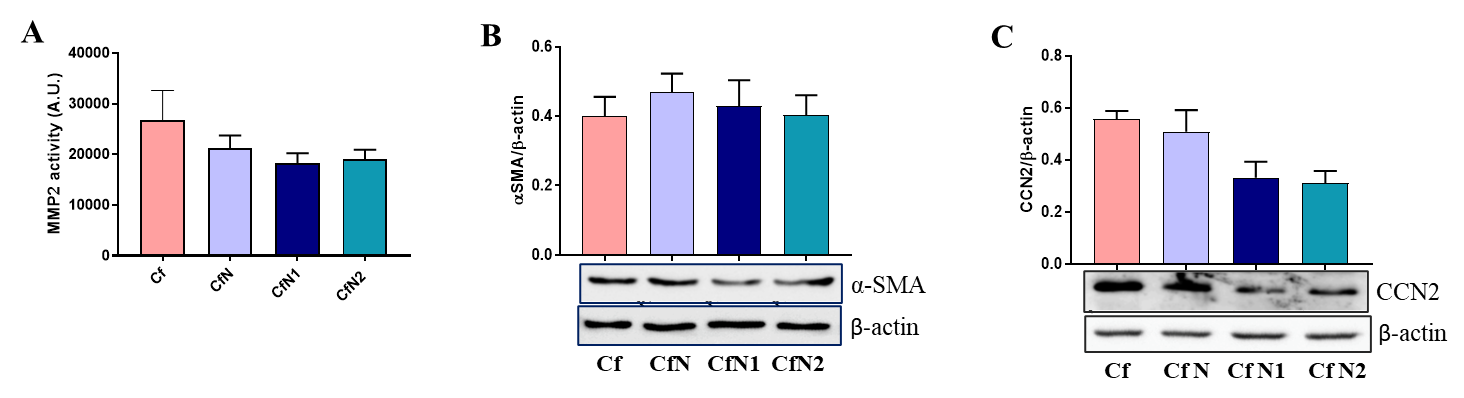


**Supplementary Figure S3**. (A) The enzymatic activity of gelatinases MMP‐2 in the conditioned media of human CFs following indirect interaction with N/N1/N2 neutrophils, assessed by SDS‐PAGE gelatine zymography; (B and C) Protein expression of myofibroblast marker α-SMA and pro-fibrotic matricellular protein CCN2 in human CFs following indirect interaction with neutrophils, as assessed by Western Blot and normalized to β-actin; n = 3 Data is represented as mean ± SEM.

**
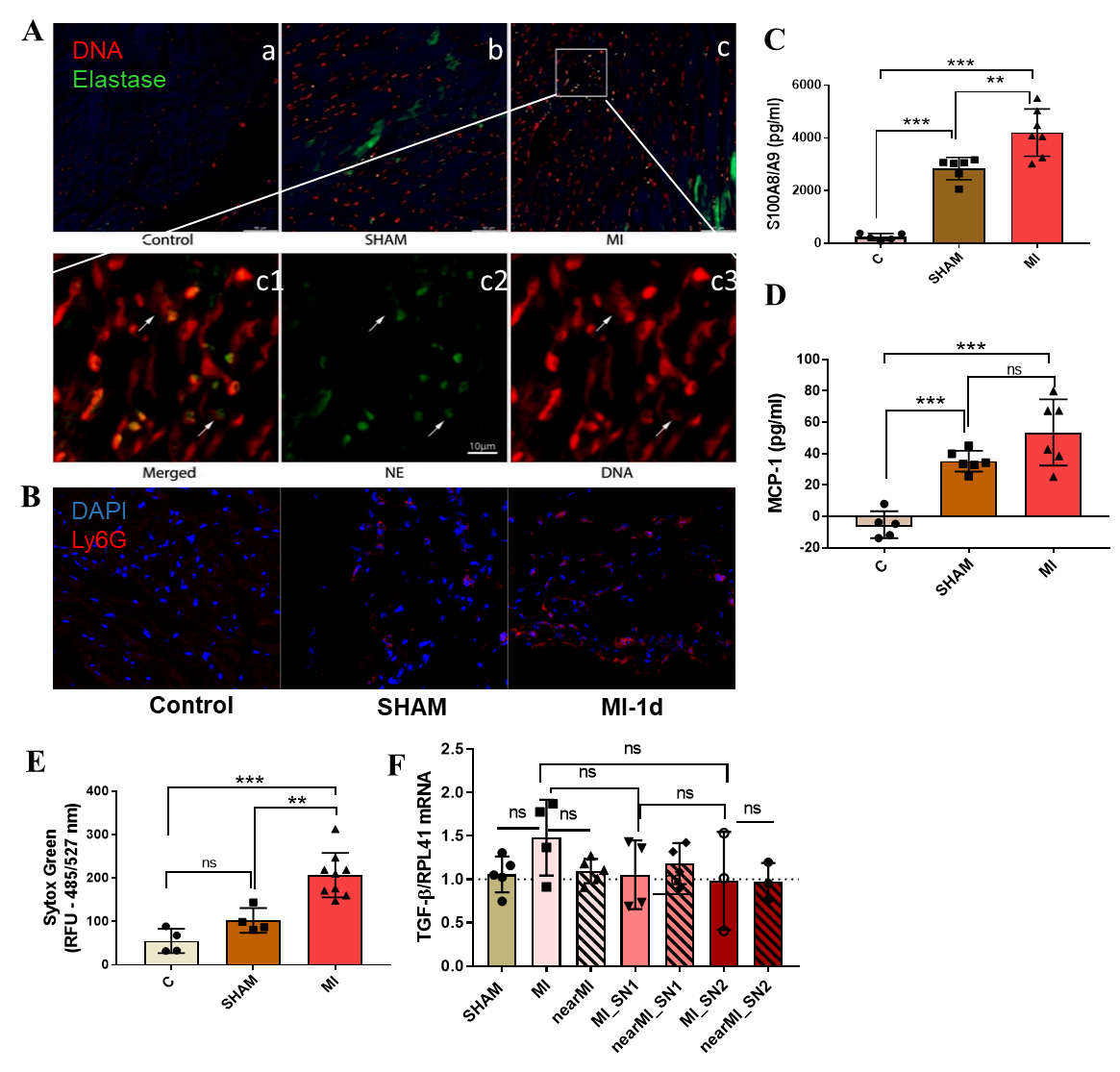
**

**Supplementary Figure S4**. Local and systemic markers of neutrophil activation in acute (day 1) MI. (**A).** Representative images of neutrophil elastase (green) in mouse cardiac tissue from control (a), SHAM (b), or mice with MI (c). DNA is stained red. Scale bar is 50µm for images a, b, and c, and 10µm for images c1-c3. The c1-c3 images show an enlarged area from image c where infiltrated neutrophils and potential NETs are visible (white arrows). (**B).** Immunofluorescence staining of neutrophil marker Ly6G (red) in mouse cardiac tissue from control, SHAM, or mice with MI. Nuclei are stained with DAPI (blue). (**C-D).** Plasma levels of S100A8/A9 and MCP-1 in the serum of control, SHAM, or mice with MI after 1 day, as evaluated by ELISA assay. (**E)**. DNA presence in serum of control, SHAM, or mice with MI after 1 day, after fluorescent staining with Sytox Green. n=4-6, **p<0.01, ***p<0.001. Data is represented as mean ± SD. **(F)**. TGF-β1 mRNA expression in LV tissue from SHAM and MI mice (± SN1/SN2 injection) at 1-day post-MI, normalized to healthy control mice that did not undergo surgical procedures, n=5/6 mice per group;

**
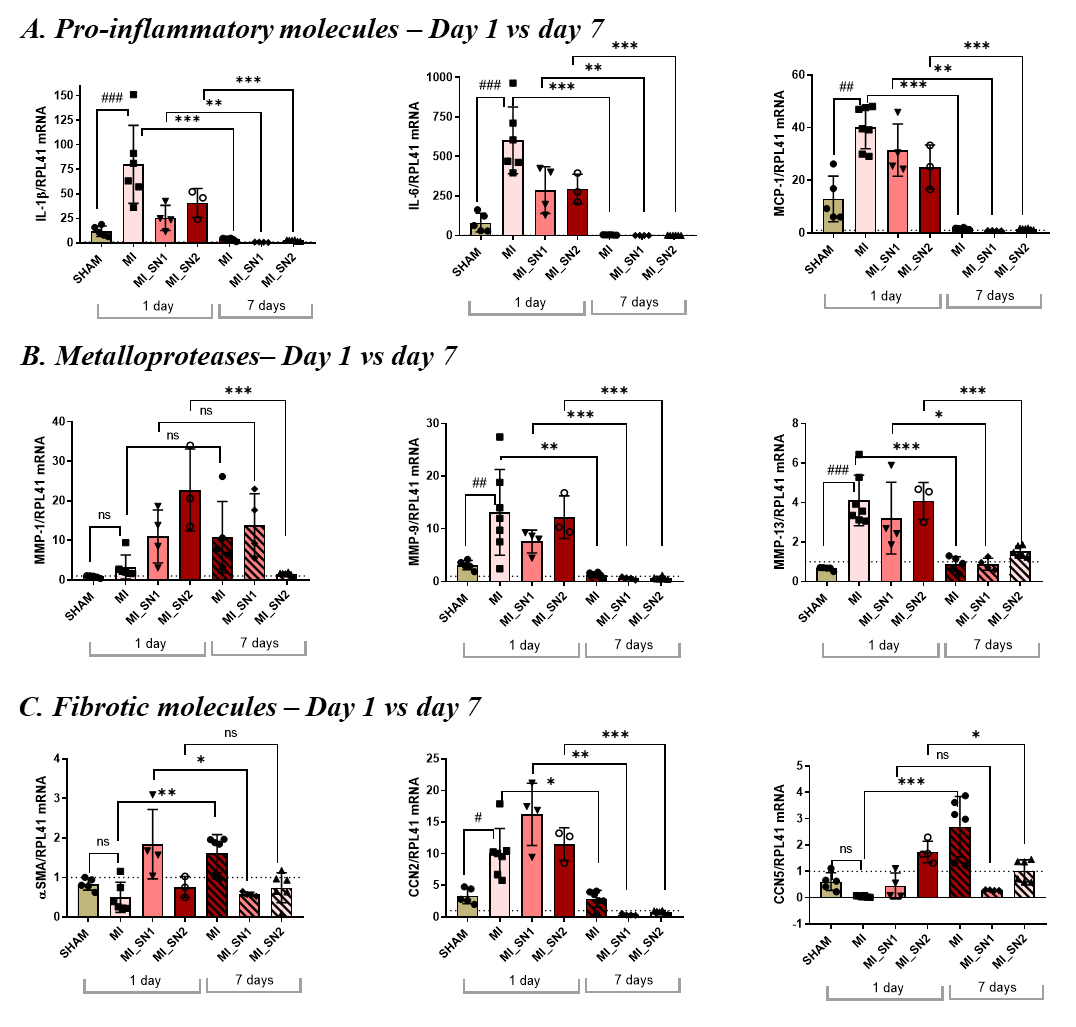
**

**Supplementary Figure S5.** Gene expression of pro-inflammatory, ECM remodelling and fibrotic mediators during the acute phase (day 1) and the reparatory phase (day 7) post-MI. (**A)**. Pro-inflammatory molecules IL-1β, IL-6, and MCP-1; (**B)**. Metalloproteases MMP-1, -9, and -13; and (**C)**. Fibrotic mediators αSMA, CCN2, and CCN5, quantified in LV tissue of SHAM, mice with MI with or without SN1/SN2. Gene expression data were generated by qPCR and normalized to healthy control mice that did not undergo surgical procedures. n=5/6 mice per group, * 1 day vs 7 days, # vs SHAM. *p<0.05, **p<0.01, ***p<0.001. Data is represented as mean ± SD.


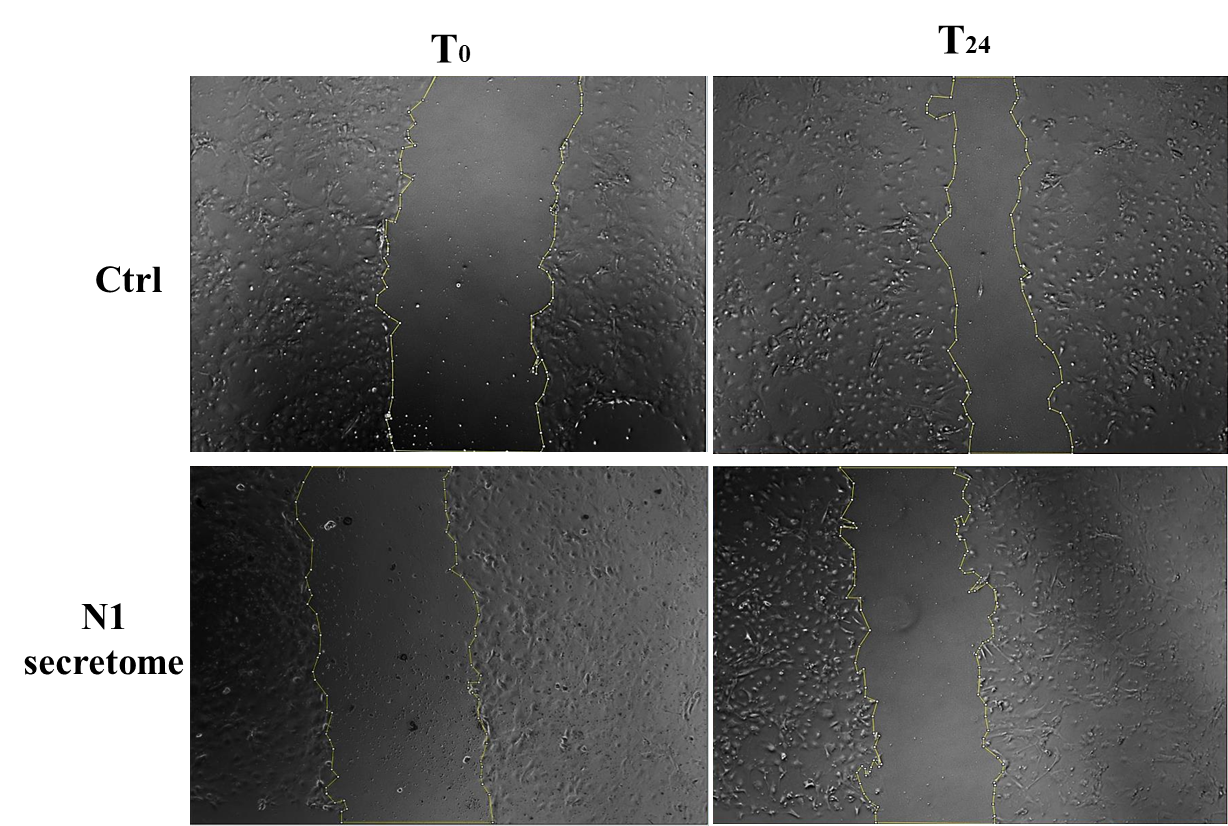


**Supplementary Figure S6.** Fibroblast migration in the presence of N1 soluble derived mediators. Representative images from *in vitro* scratch wound healing assays showing that mouse fibroblast migration is impaired by exposure to factors released by pro-inflammatory mouse BM derived neutrophils (N1 secretome) as compared with fibroblasts exposed to culture media (Ctrl) after 24h (T_24_).

**Supplementary Figure S7.** Gene expression of pro-inflammatory molecules expressed by mouse cardiac fibroblasts following interaction with mouse BM neutrophils. (**A–D).** Gene expression of inflammatory markers IL-1β, IL-6, TNFα, and metalloprotease MMP-9 in mouse cardiac fibroblasts - control (cFb), upon co-culture with neutrophils (FbN) or co-culture with neutrophils in the presence of a TLR4 inhibitor (CLI-095). n=3, **p<0.01, ***p<0.001, (cFb vs. FbN1) and #p<0.05, ##p<0.01, ###p<0.001 (FbN1 vs. FbN1+CLI).


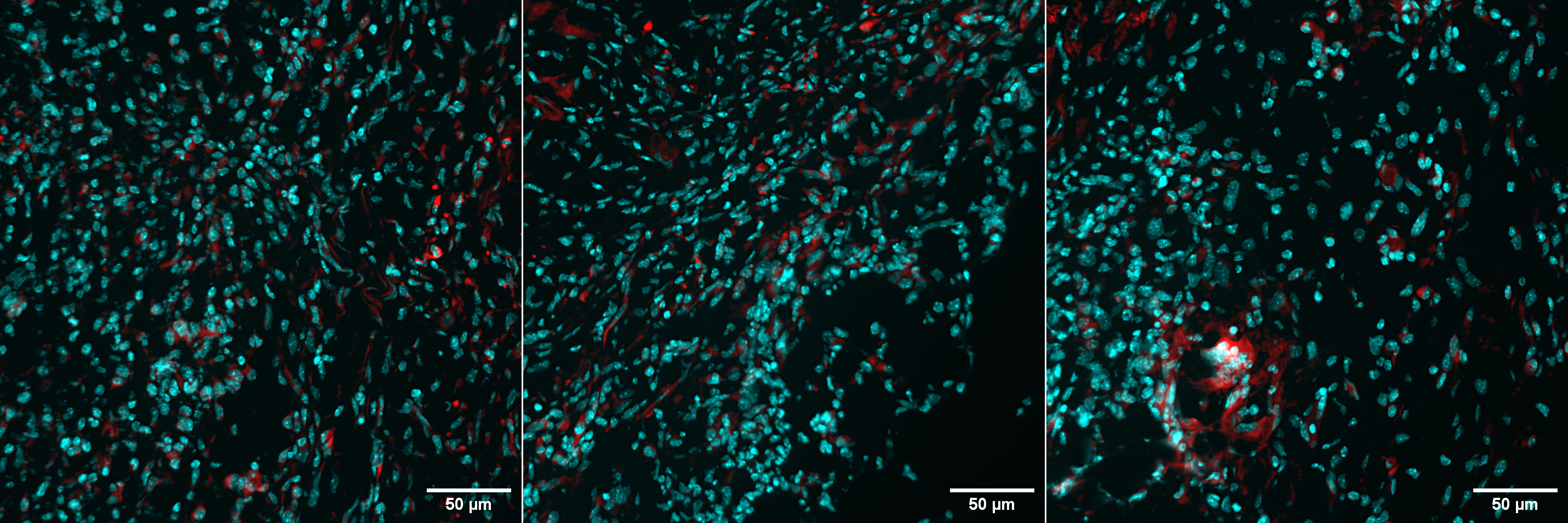


**Ki67 DAPI**

**MI**

**MI-SN1**

**MI-SN2**

**B**

**A**

**Supplementary Figure S8.** Effect of N1/N2 secretome (SN1/SN2) on cell proliferation in the reparative phase - 7 days post-MI. (A) Immunofluorescence staining of Ki67 (red) in mouse cardiac infarcted tissue from MI mice with or without treatment with SN1/SN2. Nuclei are counterstained with DAPI (cyan). Scale bar represents 50μm; (B) Signal intensity was quantified within the entire infarcted zone, selected from slide scans acquired at 20x magnification, and expressed as mean fluorescence intensity (MFI) per infarct area (a1, a2). Data is represented as mean ± SD.

**Supplementary Figure S9. DNA as a marker of NETs presence in human PMN supernatant.** Sytox green staining of DNA released in conditioned media of control (unstimulated) human PMN (N), and human PMN cultivated in the presence of N1 (LPS+IFN) and N2 (IL-4) agonists after 2.5 hours in culture. n=3, **p<0.01, ***p<0.001. Data is represented as mean ± SD control.
